# Functional and genomic characterization of a novel probiotic* Lactobacillus johnsonii* KD1 against shrimp WSSV infection

**DOI:** 10.1038/s41598-023-47897-w

**Published:** 2023-12-07

**Authors:** Kanokwan Dekham, Samuel Merryn Jones, Sarocha Jitrakorn, Patai Charoonnart, Nalumon Thadtapong, Rattanaporn Intuy, Padungsri Dubbs, Suradej Siripattanapipong, Vanvimon Saksmerprome, Soraya Chaturongakul

**Affiliations:** 1 Department of Microbiology, Faculty of Science, Mahidol University, Bangkok, 10400 Thailand; 2 School of Biosciences, Division of Natural Sciences, University of Kent, Canterbury, CT2 7NZ UK; 3 National Center for Genetic Engineering and Biotechnology (BIOTEC), National Science and Technology Development Agency (NSTDA), Pathum Thani, 12120 Thailand; 4 Center of Excellence for Shrimp Molecular Biology and Biotechnology (Centex Shrimp), Faculty of Science, Mahidol University, Bangkok, 10400 Thailand; 5 Graduate Program in Biomedical Sciences, Faculty of Allied Health Sciences, Thammasat University, Pathum Thani, 12120 Thailand; 6 Molecular Medical Biosciences Cluster, Institute of Molecular Biosciences, Mahidol University, Nakhon Pathom, 73170 Thailand

**Keywords:** Ecology, Immunology, Microbiology, Molecular biology, Zoology

## Abstract

White Spot syndrome virus (WSSV) causes rapid shrimp mortality and production loss worldwide. This study demonstrates potential use of *Lactobacillus johnsonii* KD1 as an anti-WSSV agent for post larva shrimp cultivation and explores some potential mechanisms behind the anti-WSSV properties. Treatment of *Penaeus vannamei* shrimps with *L. johnsonii* KD1 prior to oral challenge with WSSV-infected tissues showed a significantly reduced mortality. In addition, WSSV copy numbers were not detected and shrimp immune genes were upregulated. Genomic analysis of *L. johnsonii* KD1 based on Illumina and Nanopore platforms revealed a 1.87 Mb chromosome and one 15.4 Kb plasmid. Only one antimicrobial resistance gene (*ermB*) in the chromosome was identified. Phylogenetic analysis comparing *L. johnsonii* KD1 to other *L. johnsonii* isolates revealed that *L. johnsonii* KD1 is closely related to *L. johnsonii* GHZ10a isolated from wild pigs. Interestingly, *L. johnsonii* KD1 contains isolate-specific genes such as genes involved in a type I restriction-modification system and CAZymes belonging to the GT8 family. Furthermore, genes coding for probiotic survival and potential antimicrobial/anti-viral metabolites such as a homolog of the bacteriocin helveticin-J were found. Protein–protein docking modelling suggests the helveticin-J homolog may be able to block VP28–PmRab7 interactions and interrupt WSSV infection.

## Introduction

To date the trend of global shrimp production has risen from less than 75,000 metric tons to more than 5.5 million metric tons during 1980–2017 and increased continuously^1^. Especially *Penaeus vannamei* (White leg shrimp) and *Penaeus monodon* (Giant tiger prawn) are the most prominent for shrimp marketing. In Asia, *P. vannamei* is more cultivated than *P. monodon* due to specific pathogen-free broodstock, low-cost farming, and providing higher meat yields^2^. Likewise, Thailand showed production of *P. vannamei* and *P. monodon* at total 263,245 and 16,292 tons, respectively in 2014^3^. However, shrimp production is damaged by shrimp pathogens especially white spot syndrome virus (WSSV) which is a double stranded DNA virus and causing concern in Thailand due to broad infection of all penaeid species, rapid dissemination, and causing shrimp mortality 3–10 days after viral infection. Shrimps infected with WSSV show signs of symptoms including white spots, lost cuticle, reduction of feeding, and exhaustion leading to death^4^. This led to 15% loss of worldwide shrimp production and economic loss at US$ 1 billion^5,6^.

To reduce WSSV infection during shrimp cultivation, probiotics can be used to promote shrimp growth and protect against pathogens, reducing the use of antibiotics. Lactic acid bacteria (LAB) are Gram-positive and non-spore forming bacteria. They are a highly diverse and heterogenous group containing many species consisting of the genera *Lactobacillus*, *Enterococcus*, *Lactococcus*, *Pediococcus*, *Streptococcus*, *Leuconostoc*, *Carnobacterium*, and *Weissella*^7,8^. Some are also generally recognized as safe (GRAS) for use in humans and animals as probiotics. LAB can activate host immune systems, improve human and animals gastrointestinal (GI) tract immunity, and produce beneficial metabolites such as lactic acid which is an end product of carbohydrate metabolism, can inhibit pathogenic growth and can be applied to food production in humans and animals^9^.

*Lactobacillus johnsonii* (*L. johnsonii*) is a Gram positive, non-spore forming, non-motile, and facultative anaerobic bacteria^10^. Previously, *L. johnsonii* has been observed to decrease pathogenic infection and colonization, modulate host immune systems, and improve growth of animals such as pigs and poultries^11–16^. In aquaculture, *L. johnsonii* was found in the intestine of *Dicentrarchus labrax* (European sea bass) suggesting the ability of *L. johnsonii* to colonize in an aquatic animal^8,17^. However, the probiotic activities of *L. johnsonii* in aquaculture are not prevalent or clear. Thus, this study screened the role of the probiotic *L. johnsonii* KD1 in shrimp protection against WSSV and investigated shrimp immune activation. Additionally, the probiotics genome was examined to find genes potentially involved in promoting shrimp survival and pathogen inhibition for application in feed supplementation.

## Results and discussion

### Evaluation of probiotic bacteria against WSSV

**Figure 1:**
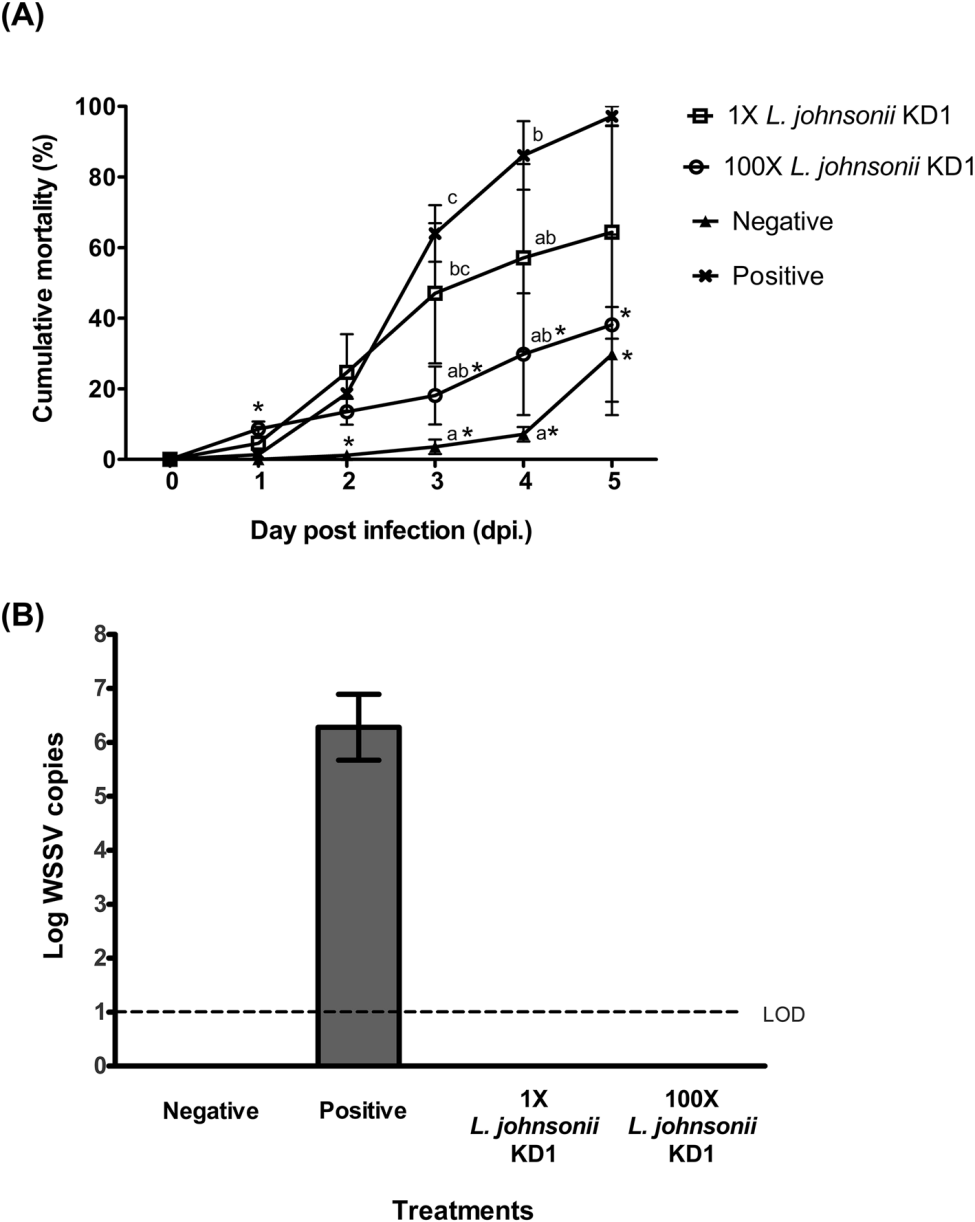
Shrimp cumulative mortality after WSSV infection (**A**) Mean of percentage cumulative mortality ± standard error in shrimps submerged with 4.5 × 10^8^ [1X] CFU/2 L seawater and 4.5 × 10^10^ [100X] CFU/2 L seawater of *L. johnsonii* KD1 for five days with n = 30 shrimps per replicate (three replicates/dose/group) while the negative and positive groups without probiotic treatments with n = 30 shrimps per replicate (three replicates/group) followed by WSSV infection except the negative group. (**B**) Mean of log WSSV copies ± standard error with 3 survived shrimps per group sampled at 3-days post infection (3 dpi.). Corresponding dpi. at p < 0.05 based on ANOVA was tested by Duncan’s test represented by small alphabets (a, b, and c). Asterisks (*) indicate significant differences between the treatment and the positive control on corresponding dpi. at p < 0.05 based on independent sample T-test and Mann–Whitney Test. Limit of detection (LOD).

Shrimps submerged with 100X *L. johnsonii* KD1 were significantly protected against WSSV, starting at 3 days post infection (dpi.) and more evident at 4 dpi., as shrimp mortality was lower than that of shrimps receiving 1X *L. johnsonii* KD1 and shrimps not receiving any probiotics (positive control) (Fig. 1A). This was consistent with the viral load assessed by qPCR on 100 ng total DNA extracted from the tissues of survived shrimps where WSSV copies were undetectable (Fig. 1B). At 5 dpi., 100X *L. johnsonii* KD1-treated shrimps showed the lowest shrimp cumulative mortality (38%) when comparing to 1X *L. johnsonii* KD1-treated shrimps (64%) and positive control (97%), respectively (Fig. 1A). Our findings suggest that a high dose of *L. johnsonii* KD1 promoted shrimp survival after WSSV infection and the intrinsic mechanism involved in its protection efficacy remains to be explored. Similarly, *P. vannamei* treated with the same high doses of *L. lactis* and *L. plantarum* could delay shrimp mortality and reduced viral progeny after WSSV infection^6^. Additionally, consortium of probiotics *Pediococcus pentosaceus* and *Staphylococcus hemolyticus* on commercial feed fed to *P. vannamei* could decrease WSSV prevalence to less than 20%^18^. Other probiotics such as *Bacillus megaterium* and *Bacillus* PC465 used in mixing feed were reported to resist WSSV infection in *P. vannamei*^4^. This evidence supports the ability of probiotics to inhibit WSSV infection and replication.

### Shrimp immune system activation by *L. johnsonii* KD1

**Figure 2:**
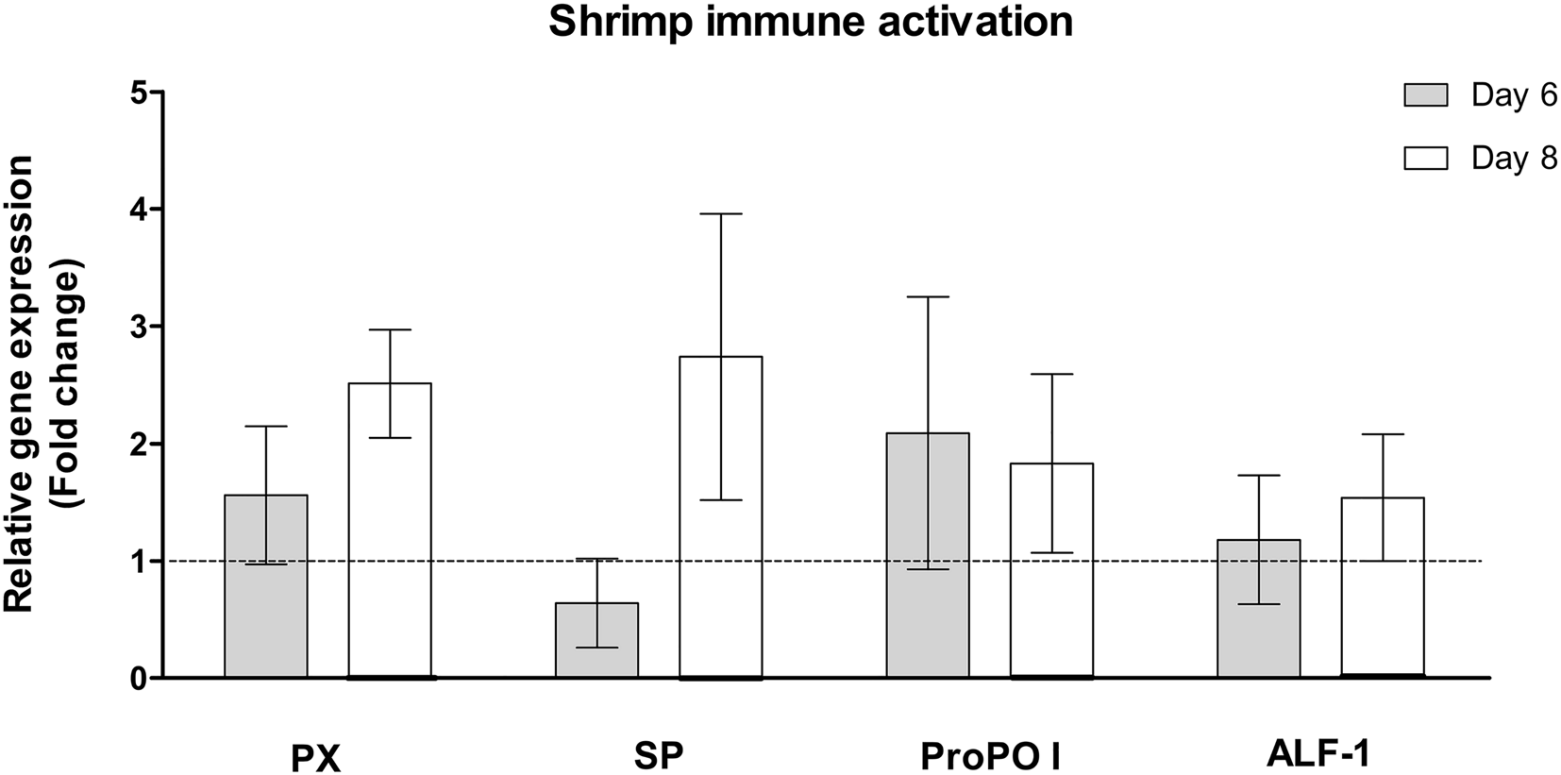
Shrimp immune gene expression of peroxinectin (PX), serine protease (SP), prophenoloxidase-1 (proPO I), and anti-lipopolysaccharide factor-1 (ALF-1). Shrimp’s regimen with 4.5 × 10^10^ [100X] CFU/2 L seawater of *L. johnsonii* KD1 for five days with n = 30 shrimps per replicate (two replicates/group) while the negative group without probiotic treatments with n = 30 shrimps per replicate (two replicates/group). Mean of fold difference of ± standard error with n = 4 shrimps per group at day 6 (or one day post-probiotics regimen in treated groups) and day 8 (or three days post-probiotics regimen). Fold difference of 1 represented as dotted line indicates no change in shrimp gene expression in probiotic treatment in comparison to without probiotic treatment (negative).

To assess *L. johnsonii* KD1 ability induce shrimp immune system, we selected four shrimp innate immune genes i.e., genes encoding peroxinectin (PX), prophenoloxidase-1 (proPO I), serine protease (SP), and anti-lipopolysaccharide factor-1 (ALF-1) and determined shrimp gene expression at day 6 (1-day post-feeding) and day 8 (3 days post-feeding) (Fig. 2). In this study, shrimps’ regimen with 100X *L. johnsonii* KD1 showed upregulation of PX at 1.6 and 2.5 folds on day 6 and day 8, respectively. Consistently, proPO I expression was increased on both days at 2.1 and 1.8 folds, respectively. SP and ALF-1 expression levels were upregulated on day 8 at 2.7 folds and 1.5 folds, respectively.

In shrimp innate immune response, PX is involved in pathogen recognition, adhesion, and eventually killing^6^ while proPO I is activated to phenoloxidase-1 by SPs and proPO-activating enzymes (PPAEs) leading to melanin production and pathogen killing^19^. Previous study reported that both PX and proPO were upregulated when *P. vannamei* was treated with *L. plantarum* in mixed feed (10^4^ and 10^7^ CFU/g feed) for 7 days^20^. Several SPs can also activate PPAEs eventually leading to proPO I induction^21^. Thus, upregulation of PX, proPO I, and SP in shrimps receiving 100X *L. johnsonii* KD1 may potentially eliminate WSSV leading to reduced viral load and infection in exposed shrimps. For ALF-1 expression, previous study showed that *Marsupenaeus japonicus* (kuruma shrimp) treated with *L. lactis* D1813 in mixed feed (10^5^ CFU/g feed) for 7 days exhibited an upregulation after 3 days and 6 days post-feeding^22^. ALF-1 is an antimicrobial peptide produced through the Toll pathway in penaeid shrimp. ALF-1 can bind WSSV proteins and block WSSV infection^23^. Thus, upregulation of ALF-1 expression in 100X *L. johnsonii* KD1-treated shrimps may potentially prevent WSSV infection.

### Genomic characteristics of *L. johnsonii* KD1

**Figure 3:**
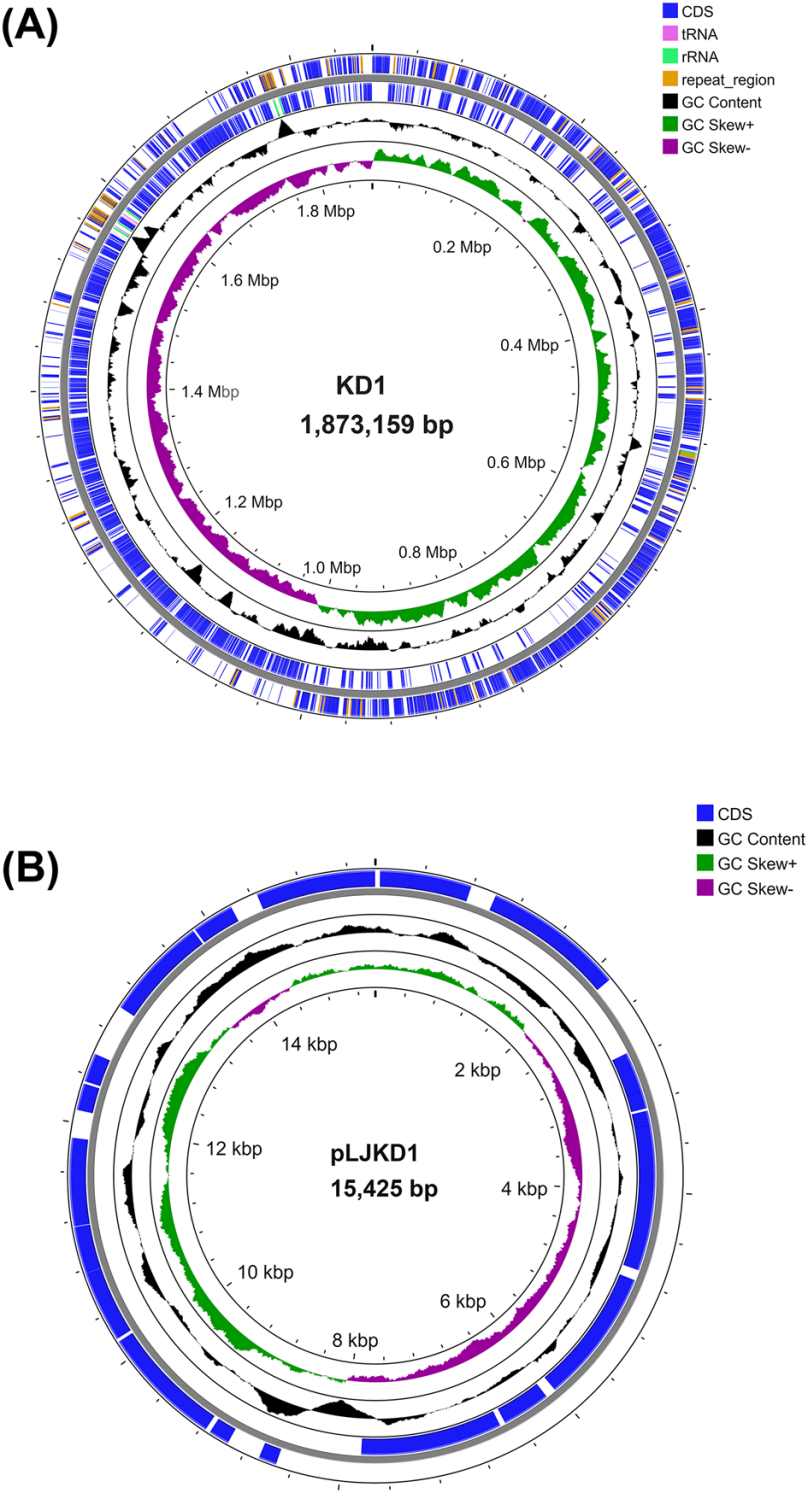
Complete genome of *L. johnsonii* KD1 (**A**) Map of chromosome (**B**) Map of plasmid.

The complete genome of *L. johnsonii* KD1 was found to consist of one 1,873,159 bp (approximately 1.87 Mb) chromosome named KD1 and one 15,425 bp plasmid named pLJKD1 with 35% and 34% GC content, respectively (Fig. 3). BLASTn of the plasmid exhibited 100% identity to *L. johnsonii* GHZ10a’s plasmid, and 94% identity to plasmids pUMNLJ21 of *L. johnsonii* UMNLJ21, pUMNLJ22 of *L. johnsonii* UMNLJ22, and pLJPF01S of *L. johnsonii* pf01, respectively. *L. johnsonii* KD1 contains 1,783 coding sequence (CDS) genes consisting of 1,390 functional genes and 393 genes encoding hypothetical proteins. Additionally, 12 genes encoding rRNA and 74 genes encoding tRNA were found. Eighty-seven genes encoding repeat regions and 9 transporter genes were identified and summarized in Table 1. For plasmid pLJKD1, there are 21 CDS genes consisting of 10 functional genes and 11 genes encoding hypothetical proteins (Table 1, Supplementary Table 1). Genes of the *L. johnsonii* KD1 chromosome and plasmid were classified into subsystems using the PATRIC database with the main subsystems of protein processing, metabolism, DNA processing and energy identified in the chromosome (Supplementary Fig. 1A) and the main subsystems of energy and stress response, defense and virulence in the probiotic plasmid (Supplementary Fig. 1B). Similarly, among *L. johnsonii* strains with probiotic activities of pathogenic inhibition and modulation of immune systems in animals such as FI9785, N6.2, ZLJ010, and BS15 showed genomic sizes of 1.76–2.11 Mb with GC content at 35% and CDS genes ranged from 1,718 to 1,959 genes^24^.

**Table 1:** Genomic characteristics of *L. johnsonii* KD1.

Features	Chromosome	Plasmid
Genome length (bp)	1,873,159	15,425
GC content (%)	35	34
L50	1	1
N50	1,873,159	15,425
Coding sequences (CDS)	1783	21
Functional proteins (genes)	1390	10
Hypothetical protein (genes)	393	11
rRNA	12	–
tRNA	74	–
Repeat region	87	–
Transporter	9	–

### Identification of an antimicrobial resistance (AMR) gene

The AMR gene *ermB* (99–100% identity) was identified at position 1,623,992–1,624,729 in the probiotic chromosome by ResFinder-4.1 (Table 2). The genes *ermT*, *ermA*, *ermB*, *ermY*, *erm46*, and *ermR* producing the Erm 23S ribosomal RNA methyltransferase have been reported to cause resistance to lincosamide, macrolide, and streptogramin b antibiotics^25^. Especially, *ermB* is prevalent in *Lactobacillus* and confers erythromycin resistance^26^. This corresponds with another study that identified *ermB* dependent erythromycin resistance in *L. johnsonii*^27,28^. Thus, antibiotic resistant profiles of *L. johnsonii* KD1 should be further investigated to confirm AMR phenotype according to European Food Safety Authority (EFSA) guideline for safety assessment for probiotic application in feed additives.

**Table 2:** Antimicrobial resistance (AMR) gene identified in *L. johnsonii* KD1.

Types	AMR genes	Positions	Lengths	Functions	Drug resistance
Chromosome	*ermB*	1,623,992–1,624,729	738 bp	23S rRNA (adenine(2058)-N(6))-methyltransferase Erm(B)	Lincosamide, Macrolide, Streptogramin b
Plasmid	*–*	*–*	–	*–*	–

### Mobile genetic elements (MGEs) and pathogenicity

To validate prophages, PHASTER was used for analysis and showed three regions of prophages in the probiotic chromosome (Table 3, Supplementary Table 2). Coding sequences of prophages showed genes encoding phage proteins, transposases, N-acetylglucosaminyltransferases, a methyltransferase, a phosphoesterase, a hydrolase, transcriptional regulators, an ABC transporter, and hypothetical proteins. Likewise, 24 insertion sequences and 5 composite transposons without association to antimicrobial resistance and virulence factors were identified by MGEfinder (Table 3, Supplementary Table 3). This suggests there will be little dissemination of antibiotic resistance and virulence factors into the environment by *L. johnsonii* KD1 mobile genetic elements. Additionally, prediction of pathogenicity by PathogenFinder 1.1 showed no pathogenic genes. *L. johnsonii* KD1 is predicted as a non-human pathogen.

**Table 3:** Mobile genetic elements identified in *L. johnsonii* KD1.

Mobile genetic elements	n	AMR genes	Virulence factors
Plasmid	1	–	–
Prophages	3	–	–
Insertion sequences	24	–	–
Transposons	5	–	–

### Identification and phylogenetic analysis of *L. johnsonii* KD1

**Figure 4:**
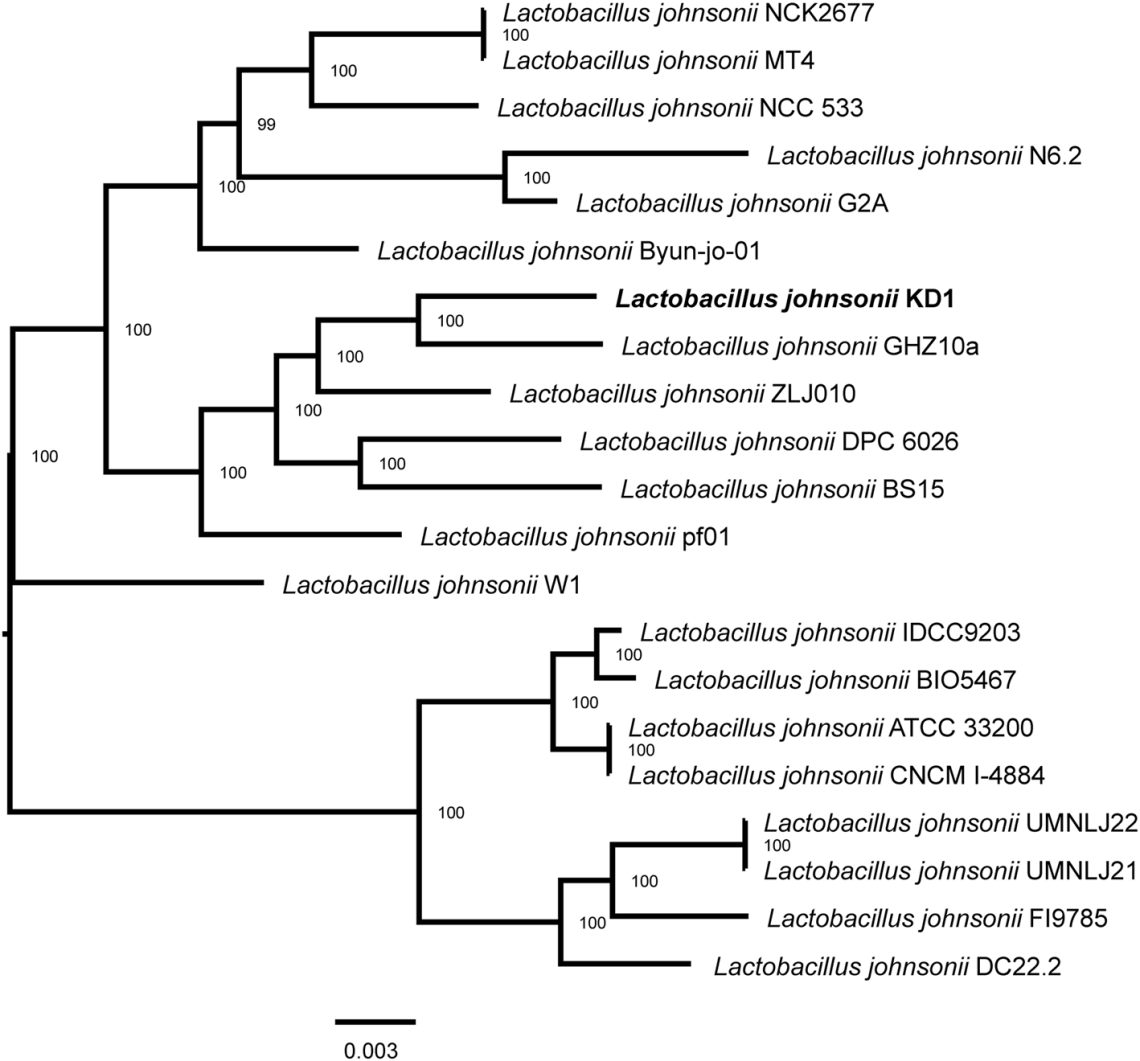
Phylogenetic tree of *L. johnsonii* strains based on 500 single-copy genes by RAxML in Phylogenetic Tree Service of BV-BRC version 3.25.3.

As the isolation source of *L. johnsonii* KD1 was unknown, we confirmed from rMLST analysis that the probiotic shares 98% identity with *L. johnsonii* (Supplementary Table 4). We then proceeded with phylogenetic analysis based on 500 single-copy genes by comparing *L. johnsonii* KD1 to 21 other *L. johnsonii* isolates in the NCBI database. The results showed that *L. johnsonii* KD1 was more closely related to *L. johnsonii* GHZ10a isolated from *Sus scrofa* or common wild pig and *L. johnsonii* ZLJ010 isolated from Sow feces^24^ (Fig. 4). We could only best suggest that *L. johnsonii* KD1 could come from swine origin.

### Comparative genomic analysis

Comparison of the genomes of *L. johnsonii* KD1 and 21 *L. johnsonii* strains identified the pan genome of 4045 genes consisting of 1065 core genes, 1932 accessory genes and 1048 strain specific genes. *L. johnsonii* KD1 showed nine unique genes encoding for late competent protein COMEA (a DNA receptor), an uncharacterized deacetylase, an integrase, hypothetical proteins, and proteins belonging to the type I restriction-modification system (Supplementary Table 5). Restriction-modification (RM) systems are part of bacterial innate immune systems and work to protect host DNA by preventing cleavage via DNA methylation and to cleave foreign DNA by the action of restriction nucleases (REases)^29–31^. They are classified into type I-IV RM systems, a type I RM system contains (1) a specificity subunit S to recognize a specific DNA motif, (2) two methyltransferase subunit M to methylate DNA, and (3) two restriction endonuclease subunit R to cleave unmethylated DNA. This type cleaves specific sequences far away from a recognition site whereas type II and IV RM systems cleave at a recognition site. Type III RM system cleaves DNA sequences similarly but lack a specificity subunit S^29^.

Previously*, L. johnsonii* DPC6026 presented a type III RM system and CRISPR elements that provided phage resistance^32^. In this study, we found genes coding for a type I RM system (Supplementary Table 6) and one CRISPR element consisting of three spaces and four direct repeats in *L. johnsonii* KD1 (Supplementary Table 7). This therefore suggests that *L. johnsonii* KD1 may exhibit phage resistant properties, however, additional experiments are required to investigate this. Due to the fact that phage contamination can be found in food and feed fermentation in non-sterile environments and could lead to low quality and loss of production^33^, using phage-resistant probiotics could bypass the problems and benefit overall food and feed productions.

As *L. johnsonii* KD1 in this study provided better anti-WSSV activity than *L. plantarum* ATCC 14917 according to a previous study^6^ (Supplementary Fig. 2), genes of *L. johnsonii* KD1 were compared to two genomes of *L. plantarum*. The comparative pathways tool identified 11 *L. johnsonii* KD1 specific pathways classified into 4 classes: xenobiotics biodegradation and metabolism*,* lipid metabolism, biosynthesis of secondary metabolites and biosynthesis of polyketides and non-ribosomal peptides. The pathways identified were incomplete, with only one or two enzymes present. However, some of products of the enzymatic reactions identified in the pathways have potential anti-viral properties. These are: Glycolate (acetic acid), 4′-hydroxyacetophenone (anti-hepatitis B activity^34^), 13-OXDE (analogue of Linoleic acid, which is active against WSSV^35^), and Taxol (HIV-1 pseudovirus inhibition^36^), (Supplementary Table 8). Then, we found 582 unique genes in *L. johnsonii* KD1 such as genes encoding type I restriction modification systems, levansucrase, a bacteriocin ABC-transporter with ATP-binding and permease component, and a bacteriocin ABC-transporter with auxiliary protein. However, 268 of the unique genes in *L. johnsonii* KD1 were identified as hypothetical. To assign function to these proteins the command-line tool MicrobeAnnotator was utilised. This annotated 44 of the 268 unique genes involved in cell wall biosynthesis, glycosylation and lipopolysaccharide biosynthesis, a variety of transporters and 16 viral proteins (Supplementary Table 9). This suggests three possibilities; (1) the proteins annotated as viral proteins share homology with viral proteins but do not share the same function; (2) the probiotic has acquired these proteins and potentially utilises them to carry out functions in the cell or 3) the strain presents viral envelope proteins on their surface as a method of hosting an immune response against the virus. This therefore suggests that different proteins/metabolites may be produced by *L. johnsonii* KD1 compared to *L. plantarum* ATCC 14917, which may provide shrimp with a higher protection to WSSV.

**Figure 5:**
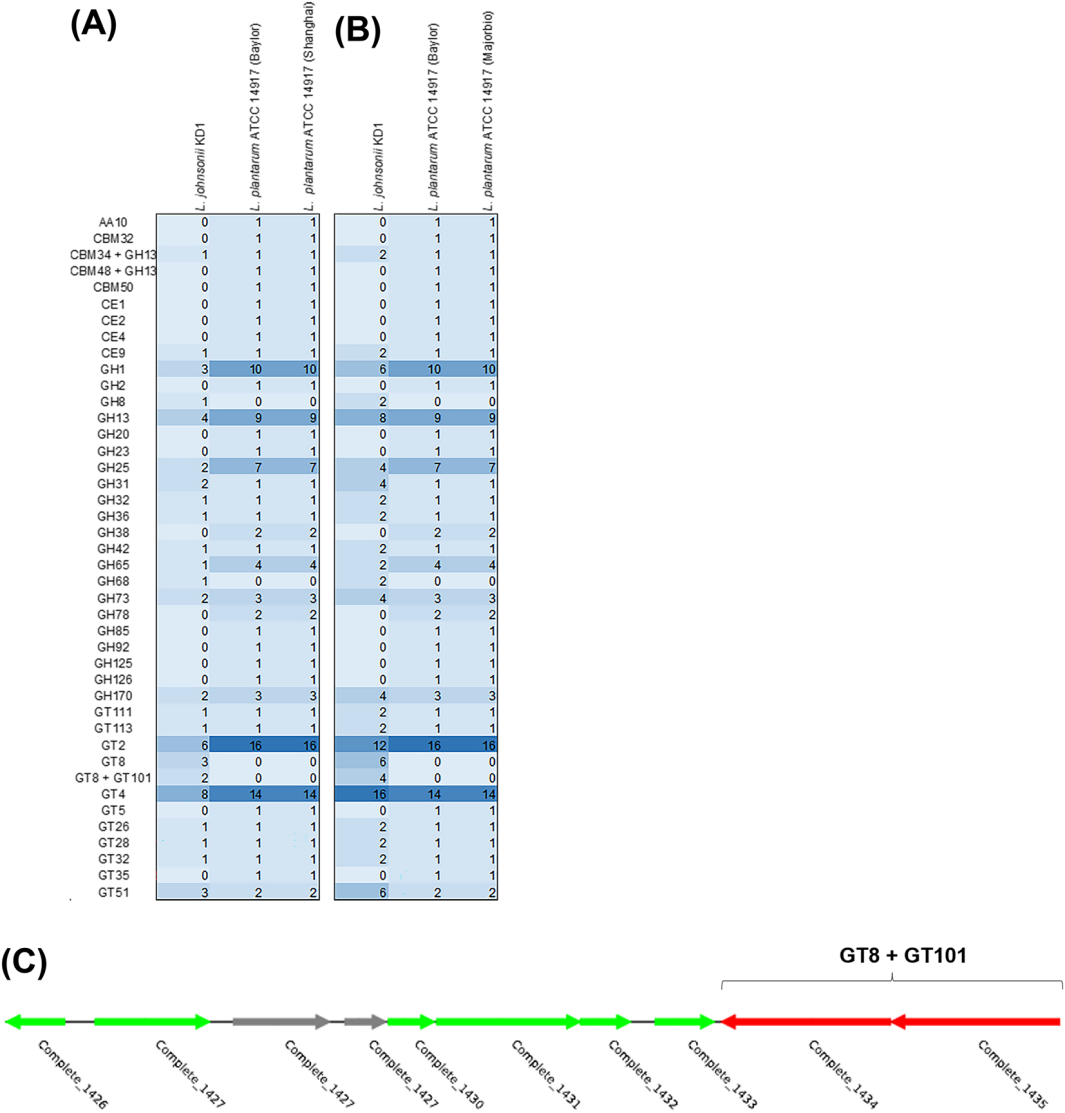
CAZymes profiles. The *L. johnsonii* KD1 and two *L. plantarum* ATCC 14917 genomes were annotated via the dbcan2 webserver. Heatmaps of (**A**) the total number of genes annotated as CAZymes in their respective families and (**B**) the percentage of each family in respect to total number of CAZymes identified in each genome were produced. (**C**) GT8 + GT101 gene containing CGC identified in the *L. johnsonii* KD1 genome (graphic generated by the dbCAN2 webserver); transporter (green), CAZyme (red), and non-signature containing/unannotated genes (grey).

Furthermore, to investigate potential differences in carbohydrate usage between the two strains, the carbohydrate active enzymes (CAZymes) were annotated using the dbCAN 2 webserver^37^. The genes can be classified as belonging to either glycoside hydrolases (GHs), glycosyltransferases (GTs), polysaccharide lyases (PLs), carbohydrate esterases (CEs), auxiliary activities (AAs) and carbohydrate-binding modules (CBMs). The classes can be further categorized based on amino acid sequence homology. As seen in Fig. 5, a variety of different families of CAZymes were identified and both *L. plantarum* ATCC 14917 genomes had identical profiles. However, when comparing *L. johnsonii* KD1 and *L. plantarum* ATCC 14917 there were differences in the relative abundances and families identified. In addition, *L. plantarum* genomes each contained twofold as many CAZymes as *L. johnsonii* KD1 (99 and 50 respectively). Interestingly, 10% of *L. johnsonii’s* CAZymes belonged to the GT8 family (6% GT8, 4% GT8 + GT101), whereas none were identified in *L. plantarum* ATCC 14917 (Fig. 5A,B). Activities associated with GT8s are lipopolysaccharide, inositol, homogalacturonan, UDP-GlcA: xylan and UDP-Gal:glucoside glycosyltransferases. Another application of the dbCAN2 webserver annotation is the identification of CAZyme gene clusters (CGC’s) which are clusters of genes which work together to digest or utilise carbohydrates^38^. One of the gene clusters identified in *L. johnsonii* KD1 contained the two GT8 + GT101 genes and 6 transporter genes, which could present a method of lipopolysaccharide biosynthesis and export (Fig. 5C). The differences observed between the CAZymes profiles of *L. johnsonii* KD1 and *L. plantarum* ATCC 14917 genomes demonstrates a difference in carbohydrate usage between the two strains. Furthermore, the appearance of GT8s only in *L. johnsonii* KD1 suggest this class of enzyme could be important in exerting the probiotic affect observed. In addition, as the GT8 family contains enzymes associated with lipopolysaccharide biosynthesis, the surface of *L. johnsonii* KD1 may display different lipopolysaccharides to *L. plantarum* ATCC 14917, which may have probiotic properties^39^. Moreover, the putative gene cluster (Fig. 5C) identified in the genome of *L. johnsonii* KD1 suggests a potential carbohydrate modification and export mechanism. Further work could be carried out investigating this pathway and its potential link to the probiotic influence of the strain.

### Probiotic survival in shrimp water cultivation and gastrointestinal tract (GI) condition

Probiotics are exposed to salt stress in the water during feeding and by bile salts in the shrimp GI produced by the shrimp hepatopancreas, which can be fatal to some bacteria. Previously, probiotics, especially LABs, have been seen to resist salinity and bile salts^40^. Accordingly, the genomes of the salt resistant strains of *L. plantarum* (D31 and T9) contained salt resistant genes such as genes coding for sodium/proton (Na^+^/H^+^) antiporters responsible for regulating intracellular pH by sodium efflux out of the cell, a potassium transport system protein, Kup, involved in ion homeostasis during hyperosmotic stress and the transcription factor LysR and RNA polymerase sigma factor RpoD involved in intracellular metabolism homeostasis during salt stress^41^. In this study, four genes encoding Na^+^:H^+^ antiporters, two genes encoding two potassium transport system proteins Kup and two transcription factors; one from the LysR family and the RNA polymerase sigma factor RpoD were identified in *L. johnsonii* KD1 (Supplementary Table 10). Thus, presenting mechanisms of *L. johnsonii* KD1 resistance to salt stress. Furthermore, *L. johnsonii* KD1 revealed three genes coding for choloylglycine hydrolases or bile salt hydrolases (Supplementary Table 10). Bile salt hydrolases deconjugate bile salts leading to release of an amino acid group from steroid core, which has been demonstrated to promote bacterial colonization and survival in the GI tract^42^. For example, *L. acidophilus* NCFM contains genes coding for bile salt hydrolases and exhibited bile salt hydrolase activity^43^. Likewise, *L. johnsonii* ZLJ010 and *L. johnsonii* CNCM I-4884 showed genes coding for the Na^+^:H^+^ antiporters and choloylglycine hydrolases. Especially, *L. johnsonii* CNCM I-4884 presented strong bile salt hydrolase activity^24,44^.

Additionally, heat shock proteins and Clp proteases can repair and refold damaged proteins in a response to stress. Moreover, the UvrABC system protein B and ATP-dependent DNA helicase UvrD/PcrA have been reported to be involved in DNA repair^4,45^. From this study, the probiotic *L. johnsonii* KD1’s genome showed genes coding for heat shock proteins, Clp proteases, and DNA repair (Supplementary Table 10). Corresponding to previous studies that reported that *L. johnsonii* ZLJ010 and CNCMI-4884 genomes contained genes encoding heat shock proteins GroEL, DnaK, and DnaJ, and genes encoding ATP-dependent intracellular proteases ClpP^24,44^. This suggests that these genes promote *L. johnsonii* KD1 survival in shrimp ponds and shrimp gut colonization.

**Figure 6:**
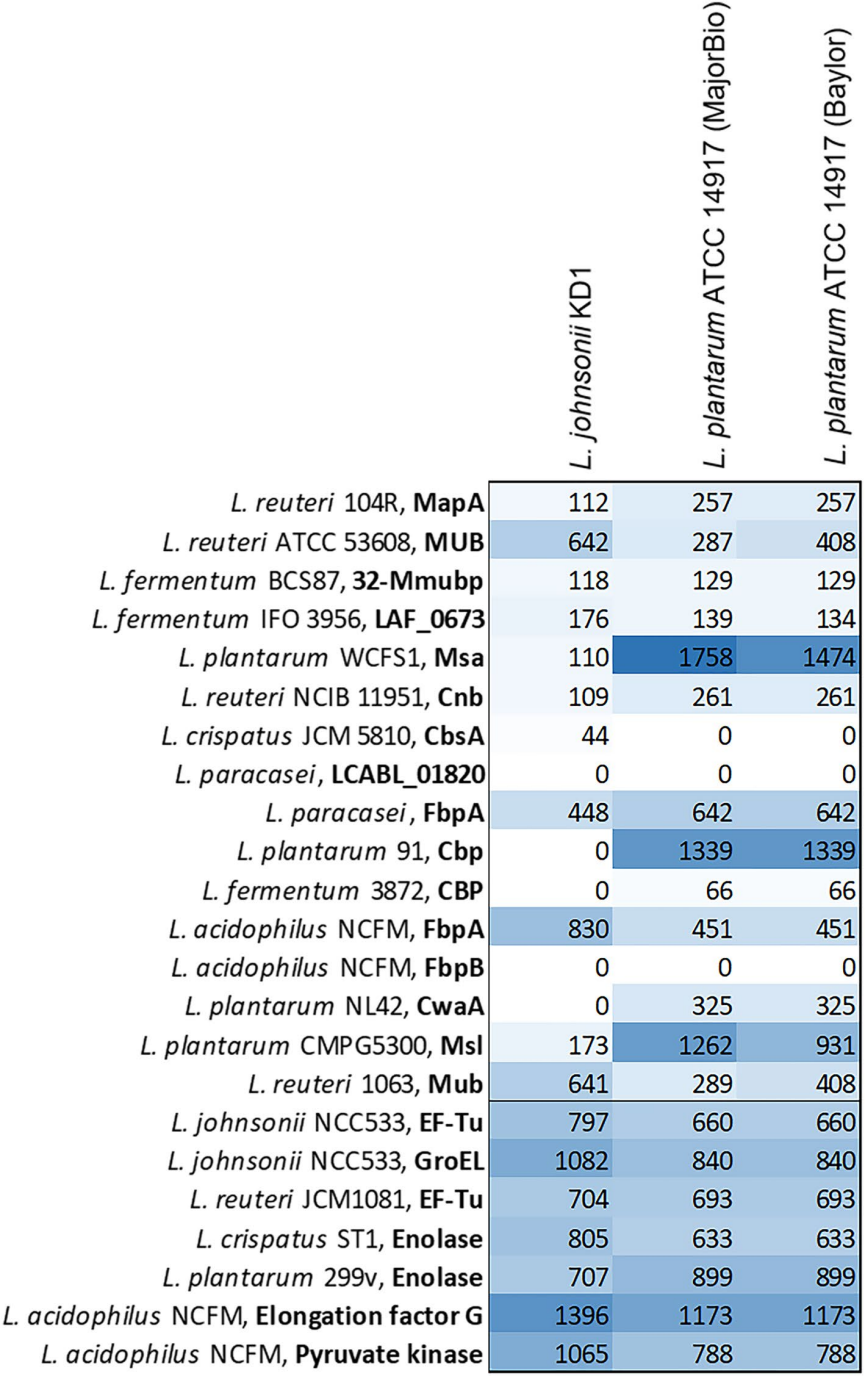
Cell-adhesion protein heat map. A heat map of cell adhesion proteins identified in the genomes of *L. johnsonii* KD1 and two *L. plantarum* ATCC 14917 was generated using the BLASTp bit-score value for the top alignment generated for each protein. Proteins above the line belong to mucus/collagen/fibronectin binding proteins. Proteins below the line belong to moonlighting cell adhesion proteins.

Another important factor for probiotics is the ability to colonise the gut of the host. This is impart mediated by the ability of the organism to adhere to the surface of gut cells. Furthermore, a possible probiotic anti-viral mechanism is via interfering with viral attachment and entry by competing for cell binding sites^46^. Over recent years some of the cell-surface proteins required for this adhesion have been assessed, thus allowing for a list of cell-adhesion proteins identified in *Lactobacillus* species to be generated from previous studies^47,48^. The presence of these proteins/homologs in *L. johnsonii* KD1 and the two *L. plantarum* genomes was then assessed using the BV-BRC BLASTp tool. A heatmap showing the bit-score value of the top hit generated by the BLASTp was produced (Fig. 6). From the cell-adhesion proteins tested, *L. plantarum* strains contain more cell adhesion proteins, thus suggesting that these *L. plantarum* strains have a higher ability to adhere to the cells of the gut and that the antiviral properties of *L. johnsonii* KD1 are not due to the better ability of the strain to adhere to the cells of the gut to physically block viral infection. However, only a selection of adhesion proteins was tested in this experiment, meaning *L. johnsonii* KD1 may contain other adhesion proteins not identified here. Future experiments could be carried out to explore the affinity of the different strains to gut cell types and localisation/surface coverage of each strain in the gut of the shrimp. This would allow for a better understanding of each strains ability to adhere to the gut of shrimp and its potential link to the antiviral probiotic affect exhibited by *L. johnsonii* KD1.

### Metabolites with potential anti-viral activities

Previously, metabolites such as lactic acid, exopolysaccharides, and antimicrobial compounds have been shown to inhibit viruses^49^. Lactic acid, a final product of carbohydrate metabolism, plays a role in the innate immune systems of, and is present in all, *Lactobacillus*. Previous studies reported that lactic acid isomer D and L could inhibit human immunodeficiency virus type 1 (HIV-1) and human simplex virus type 2 (HSV-2) replication^50–53^. In this study, we found genes encoding d-lactate dehydrogenase, responsible for d-lactate production and l-lactate dehydrogenase, responsible for l-lactate production (Supplementary Table 10). Both these isomers are the conjugate base of lactic acid, suggesting, the probiotic *L. johnsonii* KD1 can produce d-lactate and l-lactate that may potentially inhibit WSSV.

Secondly, LAB-produced exopolysaccharides (EPSs) are natural polymers of sugars/ carbohydrates and are involved in adhesion to the host GI tract for colonization, biofilm formation, and antiviral activities such as inhibition of viral infection and replication in Human Adenovirus Type 5 and rotavirus^24,49,54,55^. In this study, we performed BLASTn of the *L. johnsonii* KD1 genome using the *eps* genes of *L. johnsonii* FI9785 as queries. This identified the core *eps* gene cluster of *epsABCDE* involved in EPS biosynthesis, consisting of LytR-transcriptional regulator, polymerization and chain length determination protein, tyrosine kinase, protein-tyrosine-phosphate phosphohydrolase, and undecaprenyl-phosphate galactosephospho-transferase, respectively. Likewise, the *glf* gene encoding UDP-galactopyranase mutase, *epsU* gene encoding oligosaccharide translocase, and five genes encoding glycosyltransferases were found (Supplementary Table 10). These were similar to *eps* gene clusters found in *L. johnsonii* FI9785^56,57^. This suggests that *L. johnsonii* KD1’s ability to produce exopolysaccharides could be a potential mechanism for inhibition of WSSV infection. Moreover, inulin is a type of EPSs that increased PO activity and reduced WSSV prevalence in *P. vannamei*^58^. Additionally, inulin increased shrimp immune response and changed gut microbiota^59^. Inulin and levan are produced by inulosucrase and levansucrase enzymes, respectively, with both enzymes belonging to the fructosyltransferases family^60^. We found that *L. johnsonii* KD1 contains a gene coding for fructosyltransferase that was annotated as levansucrase at the position of 1,288,765–1,291,003 (Supplementary Table 10). However, *L. johnsonii* NCC 533 contains a fructosyltransferase (*ftf*) gene annotated as levansucrase which was able to produce inulin^60,61^. Thus, we performed a BLASTp of this protein against the UniProt database which showed a 72.5% identity (E-value = 0) hit to levansucrase and a 54.6% identity (E value = 0) to inulosucrase. This suggests that *L. johnsonii* KD1 could produce levan or inulin which could inhibit WSSV.

**Figure 7:**
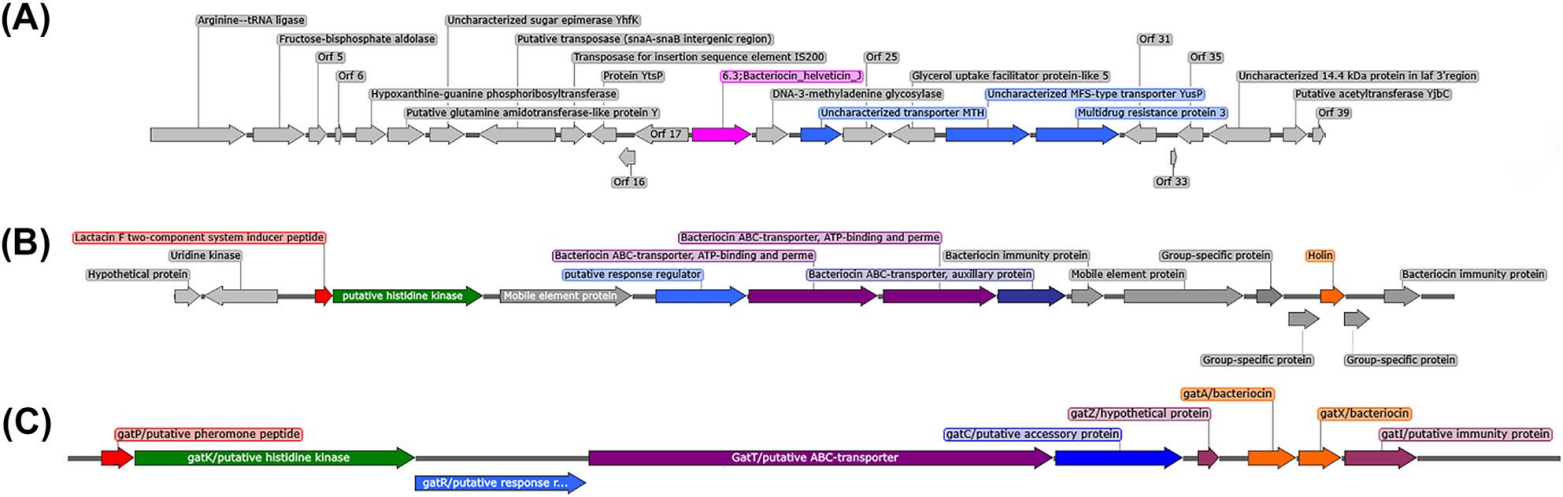
Biosynthetic gene clusters identified by antiSMASH and BAGEL4. (**A**) Bacteriocin helveticin-J homolog and gene neighbourhood identified by BAGEL4. (**B**) Holin containing gassericin T homolog cluster identified by antiSMASH. (**C**) gassericin T cluster from *Lactobacillus gasseri*. Gene annotations were produced by either antiSMASH or BAGEL4 plus further analysis using NCBI BLASTp, UniProt BLASTp and comparison to the BV-BRC annotation of *L. johnsonii* KD1's genome.

Thirdly, antimicrobial peptides such as bacteriocins have been demonstrated to inhibit viral infection, replication and modulate the host immune system^49,62^. To assess whether a potential mechanism by which *L. johnsonii* KD1 exerts its antiviral property is through the expression of a natural product, two natural product identification tools, AntiSMASH and BEGEL4, were utilized to analyse the genomes of *L. johnsonii* KD1 and *L. plantarum* ATCC 14917. This identified, in *L. johnsonii* KD1, a homolog of the bacteriocin helveticin-J originally produced by *Lactobacillus helveticus* 481 and a biosynthetic gene cluster (BGC) in which 55% of the genes had similarity to the BGC of gassericin T (Fig. 7). However, the cluster containing homology to gassericin T lacked the presence of the active peptides GatA and GatX. In addition, BLASTp analysis was unable to identify GatA or GatX homologs in the genome of *L. johnsonii* KD1. Further analysis of downstream genes identified a mobile genetic element, two bacteriocin immunity proteins and a homolog to holin. This is interesting as holins are encoded by bacteriophages to form pores in the membranes of bacteria enabling the release of endolysins to degrade the cell wall and induce death. However, studies have shown that exogenous application of holin and an endolysin from a phage called SMP can lyse pathogens such as *Staphylococcus aureus* and *Streptococcus suis*^63^. This could potentially allow for a mechanism of competing with other microbes in the absence of GatA/GatX.

BAGEL4 predicted the presence of a 352 amino acid protein, with 44.5% sequence similarity to the known bacteriocin helveticin-J. However, BLASTp of the predicted sequence against *L. johnsonii* KD1s genome identified a hit with 94% homology to the query, with the BAGEL4 prediction containing a 21 amino acid extension at the N-terminus. This is due to the BAGEL4 software predicting the start codon to be TTG (Leucine), whereas the BV-BRC RASTtk predicted the sequence to start from the downstream ATG (Methionine). Furthermore, downstream of the helveticin-J homolog are two uncharacterised transport proteins and a multidrug resistant protein, thus presenting a method of export and immunity to the action of the protein.

The holin containing gene cluster presents a potential antibacterial mechanism of *L. johnsonii* KD1. This suggests that the protein is less likely to be directly associated with WSSV inhibition and may act indirectly by influencing the gut microbiome. On the other hand, the bacteriocin helveticin-J homolog proposes a potential direct WSSV inhibition mechanism, as bacteriocins with anti-viral properties have been observed.

One mechanism by which bacteriocins can exert their antiviral properties is via blocking host cell receptor sites and preventing viral entry^64^. The ability of the helveticin-J homolog to interfere with VP28 mediated cell entry was assessed via protein–protein docking. To be able to model these potential interactions, 3D structures of the proteins were needed. Structures of the monomer and trimer of VP28 (both determined by X-ray crystallography) were downloaded from Phyre2 and RCSB Protein Data Bank (RCSB PDB), respectively. Furthermore, the AlphaFold protein structure database contained an AlphaFold V2 generated model of a protein with 100% homology to the BV-BRC identified helveticin-J homolog (Lj_peg_533) named Q74KQ5. In addition, two models of the BAGEL4 helveticin-J homolog (Lj_6.3) were generated using the google collab AlphaFold2 software, one using a PDB70 model and one without a model (Lj_6.3.1 and Lj_6.3.2, respectively). Both models were similar with an RMSD of 0.003. In addition, the AlphaFold predicted structure of PmRab7 was used, as it has been demonstrated to interact with the monomer of VP28^65^.

**Figure 8:**
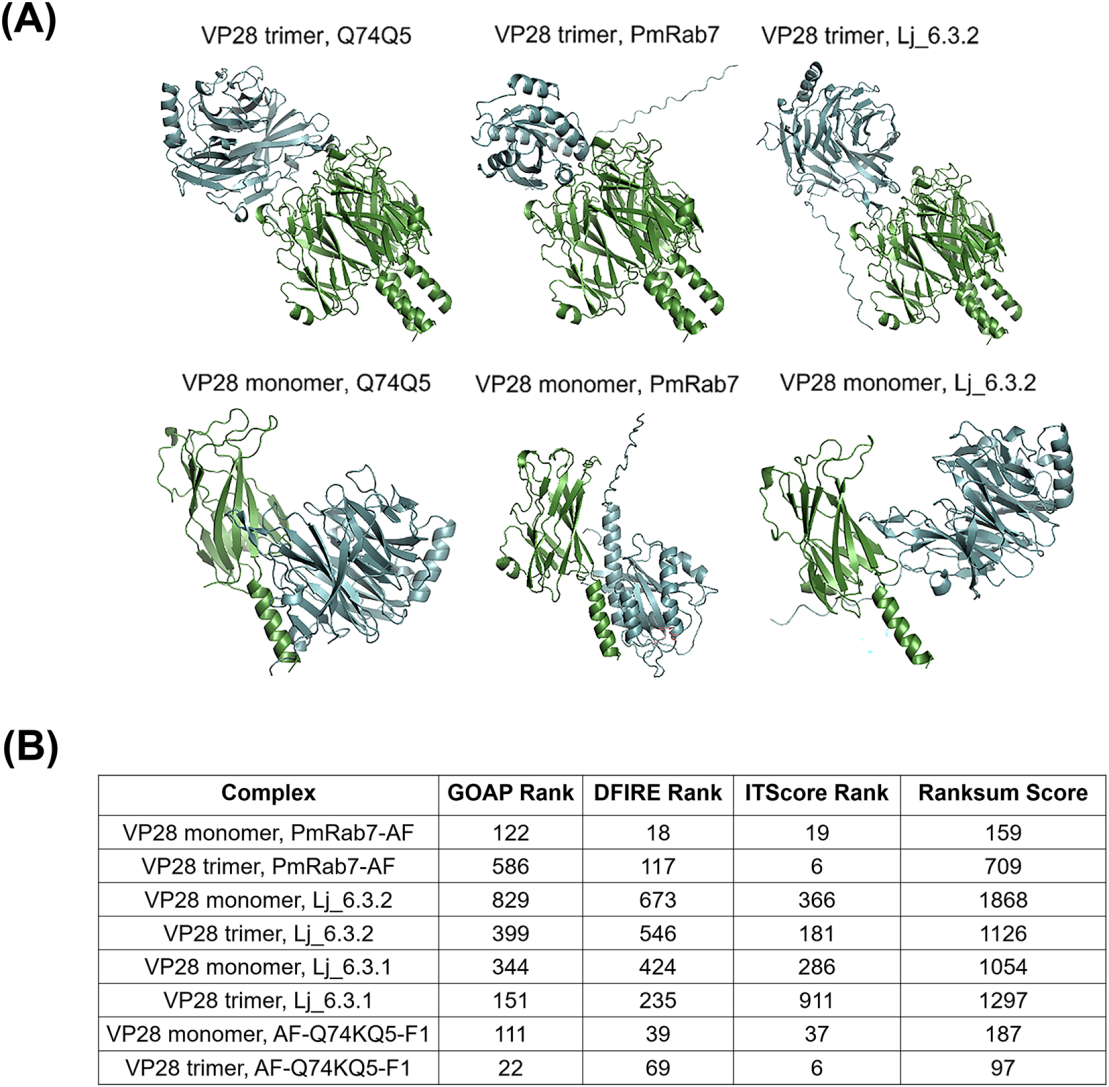
LZerD VP28 and ligand interaction models and scores. (**A**) Models produced for VP28 monomer and trimer interacting with PmRab7, Q74Q5 and Lj_6.3.2. (**B**) Individual and ranksum scores for each model.

Next, the LZerD protein docking webserver was used to generate docking models of each putative ligand (PmRab7, Lj_6.3.1, Lj_6.3.2 and Q74KQ5) with the monomer and trimer of VP28. As seen in Fig. 8, all putative ligands bind in the same region of both the VP28 monomer and trimer, thus suggesting that if these interactions were to occur then the helveticin-J homolog may be able to inhibit PmRab7 and VP28 interaction. This could therefore prevent WSSV infection, as PmRab7 binding with VP28 has been associated with WSSV infection of *P. monodon*^65^. Furthermore, *P. vannamei* has a Rab7 protein with 100% homology to PmRab7, so this interaction could be blocked in this organism too. In addition, as PmRab7 is associated with endosomal trafficking, it presents a potential different mechanism to direct inhibition of viral entry^66^. However, LZerD protein–protein docking works on the assumption that both proteins do interact. Therefore, molecular dynamics simulations should be carried out to assess the likelihood of each model of the helveticin-J homolog interacting with VP28.

Finally, this study suggests potential mechanisms behind the anti-WSSV properties demonstrated by *L. johnsonii* KD1. Thus, the list of metabolites identified in this study will be examined and tested for WSSV inhibition to identify the specific anti-WSSV mechanisms.

## Conclusions

This study investigated the roles of the probiotic *Lactobacillus johnsonii* KD1 in WSSV inhibition. Firstly, shrimps treated with 100X *L. johnsonii* KD1 reduced shrimp cumulative mortality after WSSV infection through reduction of viral production and activation of shrimp immune systems. Secondly, the complete genome of this probiotic showed one AMR gene, not related to mobile genetic elements, and a type I restriction-modification system on the probiotic chromosome. This suggests a potential phage resistance mechanism and a limited chance of AMR dissemination into the environment through mobile genetic elements. Thirdly, CAZymes analysis identified genes coding for GT8 family proteins in *L. johnsonii* KD1 which could be associated with the biosynthesis of lipopolysaccharides with potential anti-WSSV properties. Interestingly, through protein–protein docking modelling it was seen that the homolog of the bacteriocin helveticin-J identified in *L. johnsonii* KD1 may be able to block viral entry. All the findings of this study suggest that the probiotic *L. johnsonii* KD1 is friendly to use in animal supplements and able to inhibit WSSV infection. Additionally, probiotic derived metabolites could be prepared as postbiotics for shrimp cultivation in the future.

## Experimental procedures

### Preparation of probiotic supplement for application in shrimp experiments

To prepare probiotic supplement, frozen stock of *L. johnsonii* KD1 in − 80 °C freezer was streaked on De Man–Rogosa–Sharpe (MRS) agar and incubated at 37 °C without shaking for 24 h. A single colony of *L. johnsonii* KD1 was inoculated into MRS broth and incubated overnight at 37 °C without shaking. A 1:100 dilution of *L. johnsonii* KD1 culture was transferred to a new medium and incubated at 37 °C without shaking for 2.5 h. Probiotic cells at 1X (4.5 × 10^8^ CFU) and 100X (4.5 × 10^10^ CFU) were collected by centrifugation at 6000 rpm for 5 min and each probiotic pellet was resuspend in 1 mL medium prior to submersion into each shrimp tank.

### Efficacy of the probiotic *L. johnsonii* KD1 against WSSV infection in shrimp

WSSV-infected tissues were prepared according to the previous study^6^. Specific pathogen free (SPF) white shrimp *P. vannamei* juveniles (20 g B.W.) provided by Charoen Pokphand Foods (CPF) Thailand were cultured in artificial seawater with salinity of 10 parts per thousand (ppt) with aeration and average water temperature of 28 ± 0.5 °C. The juveniles were acclimatized for three days before performing experiments. Shrimps were fed with WSSV-infected tissues at 10% of shrimp body weight. Then, shrimp muscles from moribund shrimps were collected and DNA was extracted to quantify WSSV copies/g tissues by qPCR. For WSSV challenge, SPF *P. vannamei* post larvae (0.03 g B.W.) provided by CPF were cultured in artificial seawater with salinity of 20 ppt with aeration and average water temperature of 28 ± 0.5 °C. The efficacy of the probiotic treatments was tested by directly adding the probiotic into shrimp tanks. Each shrimp tank was a glass tank sized 30 × 20 × 15 cm^3^ filled with 2 L seawater. Shrimps were divided into four groups with three tanks (n = 30 shrimps/tank/2 L seawater) containing (1) shrimps’ regimen with 1X *L. johnsonii* KD1 (4.5 × 10^8^ CFU/2 L seawater), (2) 100X *L. johnsonii* KD1 (4.5 × 10^10^ CFU/2 L seawater), according to a previous study^6^, for five days before WSSV infection, (3) positive group meaning WSSV-treated shrimps without probiotic treatment, and (4) negative group meaning shrimps without any treatment (Supplementary Fig. 3A). During probiotic treatment for five days, water was not changed and then 100% water change before WSSV challenge. WSSV-infected tissues were provided to the shrimp at approximately log WSSV copies of 10.2–10.7/1 g tissues/replicate according to the previous study^6^. Numbers of live shrimps were recorded daily until 100% of mortality in the positive group. Percentage shrimp cumulative mortality was calculated from remaining percentage of cumulative shrimp survival after oral challenge with WSSV-infected tissue. Additionally, viral loads of WSSV were quantitated from three survived shrimps per group at 3-day post infection (dpi.). During experiment, the shrimps were fed with normal feed twice daily at the amount of 10% shrimp body weight per day and 100% water changes were performed every 2 days. For water quality, sufficient aeration, average water temperature of 28 ± 0.5 °C, water color, odor, pH and alkalinity were checked every day. Additionally, shrimp feces were removed every day to control water quality.

## Detection and quantitation of WSSV in shrimps by qPCR

To quantitate WSSV in shrimps, shrimp DNA was collected and extracted according to the previous study^6^. One hundred ng of shrimp DNA was used for qPCR with reactions consisting of 1X QuantiNova™ SYBR® Green PCR Master Mix (Qiagen), 0.7 µM WSSV229_F, 0.7 µM WSSV447_R primers (Supplementary Table 11) and adjusted with nuclease-free water into 20 µL final volume. Then, Rotor-Gene® Q PCR machine (Qiagen) and Rotor-Gene Q 5plex HRM Platform software were used to run qPCR and analyze the results. The viral copies were calculated from a standard curve of WSSV specific 448 amplicons at 10^9^ to 10 copies. If WSSV copies were lower than 10 copies, they were considered to be non-detectable (ND) due to limit of detection (LOD).

### Expression analysis of shrimp immune genes for probiotic treatments

To assess shrimp immune activation by probiotics we performed experiments according to the previous study^6,67^. Briefly, white shrimp *P. vannamei* post larvae (average size of 0.03 g) were treated with *L. johnsonii* KD1 at 100X dose (4.5 × 10^10^ CFU/2 L seawater) to investigate expression of shrimp innate immune genes, namely genes coding for peroxinectin (PX), serine protease (SP), prophenoloxidase-1 (proPO I), and anti-lipopolysaccharide factor-1 (ALF-1). Post-larvae shrimps were divided into two groups receiving 100X *L. johnsonii* KD1, and no bacteria (negative control). Each group contained two replicates, n = 30 shrimps/replicate/2 L seawater. Shrimps were treated with the probiotic for five days except the negative group and water was not changed during probiotic treatments. At day 6 (or day 1 post probiotic treatment) and day 8 (or day 3 post probiotics treatment), four shrimps per group were collected (Supplementary Fig. 3B). Shrimp RNA was extracted and qRT-PCR was performed according to the previous study^6^. The lists of primers are shown in Supplementary Table 11. Absolute copies of each gene were calculated based on a standard curve of 10^9^ to 10 copies of plasmids containing the shrimp immune genes. Then, each gene was normalized by a housekeeping gene coding for β-actin RNA. Finally, fold changes of shrimp gene expression were quantitated from expression ratios of shrimps treated with probiotic treatments against shrimps without probiotic treatments (negative control).

### DNA extraction of *L. johnsonii* KD1

*L. johnsonii* KD1 was cultured in 15 ml of MRS broth at 37 °C without shaking for 24 h. After that, 1.5 × 10^9^ CFU/mL was utilized for genomic DNA extraction using Exgene™ Cell SV according to GeneAll® Exgene™ manual instruction. Genomic DNA was quality checked by 0.8% gel electrophoresis and DNA concentration (ng/μL) was measured using UV absorbance at OD260/ OD280 and OD260/230 with a NanoDrop™ spectrophotometer (Thermo Scientific) and Qubit dsDNA DNA BRAssay kit (Life Technologies) followed by storage at − 20 °C until use.

### Library preparation and sequencing

One µg of genomic DNA was used to prepare a DNA library for long read sequencing by using the ligation sequencing gDNA kit (SQK-LSK110, Oxford nanopore technologies) according to the guidelines. Briefly, one µg of genomic DNA in 47 µL was added into a reaction consisting of 1 µL DNA CS, 3.5 µL NEBNext FFPE DNA Repair Buffer, 2 µL NEBNext FFPE DNA Repair Mix, 3.5 µL Ultra II End-prep Reaction Buffer, and 3 µL Ultra II End-prep Enzyme Mix followed by incubation at 20 °C for 5 min and 65 °C for 5 min. To clean up genomic DNA, 60 µL of AMPure XP beads was added and incubated for 5 min at room temperature followed by a wash with 70% ethanol. DNA pellet was dissolved with 61 µL nuclease-free water. To ligate the adapter, 60 µL DNA was mixed with 25 µL Ligation Buffer (LNB), 10 µL NEBNext Quick T4 DNA Ligase, and 5 µL Adapter Mix F (AMX-F) followed by incubation at room temperature for 10 min. Genomic DNA was cleaned up again with 40 µL of AMPure XP beads. 250 μL Long Fragment Buffer (LFB) was added. Then, DNA libraries at 5–50 fmol were loaded and sequenced by MinION and MinKNOW software v3.5.40 (Oxford Nanopore Technologies).

For short reads sequencing, 100 ng of genomic DNA was used to prepare DNA libraries for Illumina sequencing by using TruSeq DNA Nano kit (Illumina) according to TruSeq® Nano DNA Library Preparation guidelines. Briefly, 100 ng of genomic DNA was fragmented with Covaris settings. Then, DNA fragments were cleaned up with adding 80 μL SPB, 200 μL of 80% ethanol, and 62.5 μL RSB, respectively. DNA fragments was treated to blunt ends using 40 μL End Repair Mix 2 and incubated at 30 °C for 30 min followed by an incubation on ice. DNA fragments were size selected using different ratios of the SPB and performed adenylate 3ʹ ends by adding 12.5 μL ATL. To ligate adapters, 2.5 μL of each RSB, LIG2, and DNA adapters was added and cleaned up with SPB, 80% ethanol and RSB, respectively. Then, DNA fragments were enriched by adding 20 μL EPM. Finally, DNA libraries were normalized and pooled. Paired-end sequencing was performed on Illumina HiSeq platform.

### De novo assembly and annotation

Raw reads from Illumina and Nanopore were presented in Supplementary Table 12. Long reads from nanopore sequencing were concatenated by concatenate datasets tail-to-head (cat) version 0.1.1 and adaptors were removed by Porechop version 0.2.4. Long reads with a length of less than 1000 bp were removed by filtlong version 0.2.1. For short reads from Illumina sequencing, Trim Galore version 0.6.7 was used to remove adaptors from raw short reads. Then, long and short reads were combined for hybrid de novo assembly by Unicycler version 0.4.8 pipeline^68^. Complete genomes were annotated by the Rapid Annotation using Subsystem Technology toolkit (RASTtk) in the Bacterial and Viral Bioinformatics Resource Center (BV-BRC) version 3.25.3^69^ and visualized using CG View (Circular Genome Viewer) in Proksee^44^.

### Species identification and phylogenetic analysis

The chromosomal sequence was used for species determination by Ribosomal Multilocus Sequence Typing (rMLST)^70^  and for molecular typing and microbial genome diversity by PubMLST^71^. Plasmid sequence was characterized using BLASTn in National Center for Biotechnology Information (NCBI). Then, a phylogenetic tree was constructed based on 500 single-copy genes from 21 *L. johnsonii* genomes consisting of *L. johnsonii* KD1 (This study), *L. johnsonii* N6.2 (CP006811), *L. johnsonii* BIO5467 (WBNZ00000000), *L. johnsonii* Byun-jo-01 (CP029614), *L. johnsonii* FI9785 (FN298497), *L. johnsonii* G2A (CP040854), *L. johnsonii* DC22.2 (CP039261), *L. johnsonii* CNCM I-4884 (JAIQXC000000000), *L. johnsonii* NCK2677 (CP059055), *L. johnsonii* MT4 (JAJQJG000000000), *L. johnsonii* pf01 (AFQJ00000000), *L. johnsonii* ATCC 33200 (ACGR00000000), *L. johnsonii* GHZ10a (CP062068), *L. johnsonii* DPC 6026 (CP002464), *L. johnsonii* IDCC9203 (CP031701), *L. johnsonii* ZLJ010 (CP032680), *L. johnsonii* NCC 533 (AE017198), *L. johnsonii* UMNLJ21(CP021703), *L. johnsonii* UMNLJ22 (CP021704), *L. johnsonii* BS15 (CP016400), and *L. johnsonii* W1 (LSNG00000000) using Randomized Axelerated Maximum Likelihood (RAxML) in the Phylogenetic Tree Service of BV-BRC version 3.25.3^69^.

### Prediction of antimicrobial resistance, mobile genetic elements and clustered regularly interspaced short palindromic repeats (CRISPR)-Cas system, pathogenicity

Both the chromosome and plasmid sequences were investigated for antimicrobial resistance genes by ResFinder-4.1^72–74^ and CARD (Comprehensive Antibiotic Resistance Database)^75^. Additionally, prophages, insertion sequences, and transposons were identified by PHASTER (PHAge Search Tool Enhanced Release)^76,77^ and MGEfinder version 1.0.3^78^, respectively. Finally, CRISPR-Cas system was detected by using CRISPRCasFinder tool version 4.2.30^79^ and pathogenicity was predicted using PathogenFinder version 1.1^80^.

### Comparative genomics

The list of 21 *L. johnsonii* genomes as previously described were analyzed by comparative genomics using the protein family sorter program with PATRIC genus-specific families (PLfams) in BV-BRC version 3.25.3^69^ to identify core and unique coding sequences (CDS) in *L. johnsonii* KD1. Beyond comparative genomics of *L. johnsonii* strains, genomes of *L. johnsonii* KD1 and *Lactiplantibacillus plantarum*, previously named *Lactobacillus plantarum*^81^, were compared to identify unique genes of *L. johnsonii* KD1 by using the protein family sorter program with PATRIC cross-genus families (PGfams) and MicrobeAnnotator V 2.0.5 (utilising Kofam and Swiss-Prot databases)^82^. Additionally, the dbCAN2 web server^37^ was used to annotate the carbohydrate-active enzymes (CAZymes) in the *L. johnsonii* KD1 and two *L. plantarum* ATCC 14917 genomes (1 strain isolated from pickled cabbage (AZEJ00000000.1, Shanghai Majorbio) and a strain isolated from a human gut (ACGZ00000000.2, Baylor College of Medicine) using all three gene annotation tools (HMMER: dbCAN, DIAMOND: CAZy and HMMER: dbCAN-sub) plus the CGC finder tool. Annotations produced by two or more gene annotation tools were accepted. Heatmaps were then generated to compare the CAZyme profiles of each genome.

### Cellular adhesion protein identification

*Lactobacillus* cellular adhesion proteins were identified in the literature^47,48^ and sequences were obtained from NCBI^83^. The presence of cell adhesion proteins was identified in *L. johnsonii* KD1 and the two *L. plantarum* ATCC 14917 isolates using the BV-BRC BLASTp tool. Heatmaps were then generated using the bit-score of the tops hits.

### Prediction of antimicrobial peptide production

The genomes of *L. johnsonii* KD1 and *L. plantarum* ATCC 14917 were analyzed for the presence of genes encoding putative antimicrobials by BAGEL4^84^ and AntiSMASH version 6.1.1^85^. Further analysis of the genes identified surrounding the gassericin T cluster homolog was conducted using NCBI BLASTp, UniProt BLASTp and compared to the BV-BRC *L. johnsonii* KD1 annotation^86,87^.

### Bacteriocin helveticin-J homolog: VP28 interaction modelling:

Structures of the VP28 monomer and trimer (d2ed6a1 and 2ED6, respectively) were collected from Phyre2^88^ and RCSB Protein Data Bank (RCSB PDB). The structures of PmRab7 and a homolog of the bacteriocin helveticin-J from *L. johnsonii* (strain CNCM I-12250/La1/NCC 533) (Q74KQ5) (which has 100% sequence similarity to the BV-BRC predicted sequence of the helveticin-J homolog from *L. johnsonii* KD1) were obtained from the AlphaFold protein structure database^89^. Furthermore, two model structures of the BAGEL4 predicted sequence of the helveticin-J homolog in *L. johnsonii* KD1 were produced using the google collab AlphaFold V2, with a PDB70 template or no template filter selected. Structures were named Lj_6.3.1 and Lj_6.3.2, respectively. The LZerD web server^90^ was utilised to produce models of PmRab7, Q74KQ5, Lj_6.3.1 and Lj_6.3.2 interactions with the monomer and trimer of VP28. The 3D structures of respective proteins were used as inputs with the clustering cutoff (Å) set to 4 and surface reduction cutoff set to 1e-4 (default settings).

### Statistical analysis

Student’s T-test, Mann–Whitney Test, one-way ANOVA on SPSS statistics with Duncan post-hoc test were used in data analyses. Statistically significance is reported when p-value is less than 0.05.

### Ethics statement

All applicable institution guideline MUSC-IACUC, protocol no. MUSC63-003-511 and MUSC66-023-653 for the care and use of animals were followed.

### Supplementary Information

Supplementary Information.

## Data Availability

All data generated or analyzed during this study are included in this published article (and its supplementary information file). The complete genome and plasmid sequence data have been submitted to the GenBank databases under BioProject PRJNA936661 and accession numbers CP118625 and CP118626, respectively.
