# Supplementary material for: Functional and genomic characterization of a novel probiotic Lactobacillus johnsonii KD1 against shrimp WSSV infection
Source: Sci Rep. 2023 Dec 7;13:21610. doi: 10.1038/s41598-023-47897-w (PMC10703779; doi:10.1038/s41598-023-47897-w)
Supplement: Supplementary file 1 — Supplementary Information. [file 41598_2023_47897_MOESM1_ESM.docx]

**Supplementary Figures and Tables**

**Functional and genomic characterization of a novel probiotic *Lactobacillus johnsonii* KD1 against shrimp WSSV infection**

**Authors:** Kanokwan Dekham^1^, Samuel Merryn Jones^2^, Sarocha Jitrakorn^3,4^, Patai Charoonnart^3,4^, Nalumon Thadtapong^5^, Rattanaporn Intuy^1^, Padungsri Dubbs^1^, Suradej Siripattanapipong^1^, Vanvimon Saksmerprome^3,4*^, Soraya Chaturongakul^6*^

^1^Department of Microbiology, Faculty of Science, Mahidol University, Bangkok 10400, Thailand

^2^School of Biosciences, Division of Natural Sciences, University of Kent, Canterbury, CT2 7NZ, UK

^3^National Center for Genetic Engineering and Biotechnology (BIOTEC), National Science and Technology Development Agency (NSTDA), Pathum Thani 12120, Thailand

^4^Center of Excellence for Shrimp Molecular Biology and Biotechnology (Centex Shrimp), Faculty of Science, Mahidol University, Bangkok 10400, Thailand

^5^Graduate Program in Biomedical Sciences, Faculty of Allied Health Sciences, Thammasat University, Pathum Thani 12120, Thailand

^6^Molecular Medical Biosciences Cluster, Institute of Molecular Biosciences, Mahidol University, Nakhon Pathom 73170, Thailand

*** Corresponding authors:** Vanvimon Saksmerprome (vanvimon.sak@biotec.or.th) and Soraya Chaturongakul (soraya.cha@mahidol.ac.th)

**Supplementary Table 1** List of coding sequences in the pLJKD1 plasmid of *L. johnsonii* KD1

| **CDS** | **Start** | **End** | **Length** | **Product** |
| --- | --- | --- | --- | --- |
| 1 | 10185 | 10769 | 585 | Phosphoenolpyruvate-dihydroxyacetone phosphotransferase (EC 2.7.1.121), ADP-binding subunit DhaL |
| 2 | 10771 | 11154 | 384 | Phosphoenolpyruvate-dihydroxyacetone phosphotransferase (EC 2.7.1.121), subunit DhaM; DHA-specific IIA component |
| 3 | 11160 | 11876 | 717 | Glycerol uptake facilitator protein @ Propanediol diffusion facilitator |
| 4 | 12105 | 12323 | 219 | hypothetical protein |
| 5 | 12344 | 12577 | 234 | FIG00751784: hypothetical protein |
| 6 | 13006 | 13857 | 852 | site-specific recombinase, phage integrase family |
| 7 | 13876 | 14217 | 342 | Single-stranded DNA-binding protein |
| 8 | 14449 | 15423 | 975 | hypothetical protein |
| 9 | 2743 | 3102 | 360 | hypothetical protein |
| 10 | 3099 | 3257 | 159 | hypothetical protein |
| 11 | 3280 | 3411 | 132 | hypothetical protein |
| 12 | 3404 | 4705 | 1302 | hypothetical protein |
| 13 | 39 | 785 | 747 | hypothetical protein |
| 14 | 4800 | 6002 | 1203 | DNA-cytosine methyltransferase (EC 2.1.1.37) |
| 15 | 6097 | 6528 | 432 | Very-short-patch mismatch repair endonuclease (G-T specific) |
| 16 | 6578 | 6955 | 378 | replication-associated protein RepC |
| 17 | 6948 | 7838 | 891 | Chromosome (plasmid) partitioning protein ParA |
| 18 | 8527 | 8673 | 147 | hypothetical protein |
| 19 | 8951 | 9118 | 168 | hypothetical protein |
| 20 | 9160 | 10158 | 999 | Phosphoenolpyruvate-dihydroxyacetone phosphotransferase (EC 2.7.1.121), dihydroxyacetone binding subunit DhaK |
| 21 | 996 | 2144 | 1149 | Hypothetical protein SAV1803 |

**Supplementary Table 2** Prophages in the chromosome of *L. johnsonii* KD1 predicted by PHASTER

| **Prophages** | **Position and length** | **GC%** | **Total proteins** |
| --- | --- | --- | --- |
| 1. PHAGE_Stx2_c_Stx2a_F451_NC_049924  2. PROPHAGE_Pseudo_KT2440  3. PROPHAGE_Pseudo_KT2440 | 386,475-3,955,41  (9 kb) | 38.34 | 12 |
| 1. PHAGE_Stx2_c_Stx2a_F451_NC_049924  2. PHAGE_Staphy_phiN315_NC_004740  3. PHAGE_Bacill_vB_BtS_BMBtp14_NC_048640  4. PHAGE_Lactob_SAC12B_NC_048754  5. PHAGE_Faecal_FP_Lagaffe_NC_047911 | 413,208-423,869  (10.6 kb) | 36.31 | 15 |
| 1. PHAGE_Yersin_vB_YenM_TG1_NC_028820  2. PHAGE_Pseudo_PMBT14_NC_048687  3. PHAGE_Lactob_phiJB_NC_022775  4. PHAGE_Bacill_vB_BceS_MY192_NC_048633  5. PHAGE_Bacill_SPbeta_NC_001884 | 769,748-777,502  (7.7 kb) | 35.64 | 9 |

**Supplementary Table 3** Mobile genetic elements (MGEs) in *L. johnsonii* KD1’s genome as predicted by MGEfinder version 1.0.3

| **Mobile genetic elements (MGEs)** | **Family** | **Type** | **Position** |
| --- | --- | --- | --- |
| ISLjo2 | ISL3 | Insertion sequence | 839,015-840,476 |
| ISLjo2 | ISL3 | Insertion sequence | 836,506-837,967 |
| ISLjo2 | ISL3 | Insertion sequence | 19,674-21,134 |
| ISLjo2 | ISL3 | Insertion sequence | 230,704-232,164 |
| ISLjo2 | ISL3 | Insertion sequence | 79,262-80,722 |
| ISLjo2 | ISL3 | Insertion sequence | 468,494-469,954 |
| ISLjo2 | ISL3 | Insertion sequence | 1,007,822-1,009,282 |
| ISLjo2 | ISL3 | Insertion sequence | 1,768,341-1,769,792 |
| ISL4 | IS110 | Insertion sequence | 73,691-75,348 |
| ISL4 | IS110 | Insertion sequence | 705,123-706,780 |
| ISL4 | IS110 | Insertion sequence | 887,217-888,874 |
| ISL4 | IS110 | Insertion sequence | 537,005-538,662 |
| ISL4 | IS110 | Insertion sequence | 1,788,867-1,790,524 |
| ISL4 | IS110 | Insertion sequence | 186,963-188,620 |
| ISL4 | IS110 | Insertion sequence | 1,663,744-1,665,401 |
| ISL4 | IS110 | Insertion sequence | 264,441-266,098 |
| ISL4 | IS110 | Insertion sequence | 958,742-960,399 |
| ISL4 | IS110 | Insertion sequence | 774,262-775,919 |
| ISL4 | IS110 | Insertion sequence | 698,834-700,491 |
| ISL4 | IS110 | Insertion sequence | 590,269-591,926 |

**Supplementary Table 3** Mobile genetic elements (MGEs) in *L. johnsonii* KD1’s genome as predicted by MGEfinder version 1.0.3 (cont.)

| **Mobile genetic elements (MGEs)** | **Family** | **Type** | **Position** |
| --- | --- | --- | --- |
| ISL4 | IS110 | Insertion sequence | 1,383,776-1,385,433 |
| ISL4 | IS110 | Insertion sequence | 470,444-472,101 |
| ISL4 | IS110 | Insertion sequence | 584,281-585,937 |
| ISLh1 | IS982 | Insertion sequence | 982,327-983,279 |
| cn_3971_ISLjo2 | ISL3 | Composite transposon | 836,505-840,476 |
| cn_48933_ISL4 | IS110 | Composite transposon | 537,004-585,937 |
| cn_7646_ISL4 | IS110 | Composite transposon | 584,280-591,926 |
| cn_7947_ISL4 | IS110 | Composite transposon | 698,833-706,780 |
| cn_26338_ISLjo1 | IS30 | Composite transposon | 955,889-982,227 |

**Supplementary Table 4** Species identification by Ribosomal Multilocus Sequence Typing (rMLST) in public databases for molecular typing and microbial genome diversity (PubMLST)

| Locus | Allele | Length | Start position | End position | Linked data values |
| --- | --- | --- | --- | --- | --- |
| BACT000001 (*rpsA*) | 6119 | 1203 | 1124835 | 1126037 | species: *Lactobacillus johnsonii* [n=2] |
| BACT000002 (*rpsB*) | 18059 | 786 | 780654 | 781439 | species: *Lactobacillus johnsonii* [n=5] |
| BACT000003 (*rpsC*) | 4365 | 669 | 349059 | 349727 | species: *Lactobacillus johnsonii* [n=15] |
| BACT000004 (*rpsD*) | 404 | 612 | 1243862 | 1244473 | species: *Lactobacillus johnsonii* [n=22] |
| BACT000005 (*rpsE*) | 618 | 525 | 353445 | 353969 | species: *Lactobacillus johnsonii* [n=35]; *Lactobacillus* sp. [n=1] |
| BACT000006 (*rpsF*) | 350 | 297 | 8886 | 9182 | species: *Lactobacillus johnsonii* [n=54]; *Lactobacillus* sp. [n=1] |
| BACT000007 (*rpsG*) | 401 | 471 | 342746 | 343216 | species: *Lactobacillus johnsonii* [n=31] |
| BACT000008 (*rpsH*) | 3896 | 399 | 352087 | 352485 | species: *Lactobacillus johnsonii* [n=14] |
| BACT000009 (*rpsI*) | 18434 | 396 | 363229 | 363624 | species: *Lactobacillus johnsonii* [n=11] |
| BACT000010 (*rpsJ*) | 45505 | 309 | 345613 | 345921 | species: *Lactobacillus johnsonii* [n=1] |
| BACT000011 (*rpsK*) | 577 | 390 | 357422 | 357811 | species: *Lactobacillus johnsonii* [n=33] |
| BACT000012 (*rpsL*) | 632 | 408 | 342315 | 342722 | species: *Lactobacillus johnsonii* [n=45] |
| BACT000013 (*rpsM*) | 577 | 348 | 357050 | 357397 | species: *Lactobacillus johnsonii* [n=56]; *Lactobacillus* sp. [n=1] |

**Supplementary Table 4** Species identification by Ribosomal Multilocus Sequence Typing (rMLST) in public databases for molecular typing and microbial genome diversity (PubMLST) (cont.)

| Locus | Allele | Length | Start position | End position | Linked data values |
| --- | --- | --- | --- | --- | --- |
| BACT000014 (*rpsN*) | 347 | 258 | 195449 | 195706 | species: *Lactobacillus johnsonii* [n=39] |
| BACT000014 (*rpsN*) | 311 | 186 | 351877 | 352062 | species: *Lactobacillus gasseri* [n=58]; *Lactobacillus johnsonii* [n=58]; *Lactobacillus taiwanensis* [n=28]; *Lactobacillus paragasseri* [n=20]; *Lactobacillus* sp. [n=2] |
| BACT000015 (*rpsO*) | 14329 | 270 | 1186707 | 1186976 | species: *Lactobacillus johnsonii* [n=2] |
| BACT000016 (*rpsP*) | 3575 | 273 | 766871 | 767143 | species: *Lactobacillus johnsonii* [n=12] |
| BACT000017 (*rpsQ*) | 342 | 267 | 350377 | 350643 | species: *Lactobacillus johnsonii* [n=58]; *Lactobacillus* sp. [n=1] |
| BACT000018 (*rpsR*) | 752 | 237 | 9759 | 9995 | species: *Lactobacillus johnsonii* [n=52] |
| BACT000019 (*rpsS*) | 543 | 288 | 348383 | 348670 | species: *Lactobacillus johnsonii* [n=25] |
| BACT000020 (*rpsT*) | 316 | 258 | 1187171 | 1187428 | species: *Lactobacillus johnsonii* [n=20]; *Lactobacillus taiwanensis* [n=2] |
| BACT000021 (*rpsU*) | 202 | 177 | 875964 | 876140 | species: *Lactobacillus johnsonii* [n=57]; *Lactobacillus gasseri* [n=56]; *Lactobacillus taiwanensis* [n=27]; *Lactobacillus paragasseri* [n=22]; *Lactobacillus* sp. [n=2] |
| BACT000030 (*rplA*) | 22328 | 693 | 439780 | 440472 | species: *Lactobacillus johnsonii* [n=3] |
| BACT000031 (*rplB*) | 43315 | 837 | 347525 | 348361 |  |
| BACT000032 (*rplC*) | 908 | 630 | 345948 | 346577 | species: *Lactobacillus johnsonii* [n=29] |

**Supplementary Table 4** Species identification by Ribosomal Multilocus Sequence Typing (rMLST) in public databases for molecular typing and microbial genome diversity (PubMLST) (cont.)

| Locus | Allele | Length | Start position | End position | Linked data values |
| --- | --- | --- | --- | --- | --- |
| BACT000033 (*rplD*) | 243 | 618 | 346592 | 347209 | species: *Lactobacillus johnsonii* [n=32] |
| BACT000034 (*rplE*) | 592 | 543 | 351320 | 351862 | species: *Lactobacillus johnsonii* [n=33]; *Lactobacillus* sp. [n=1] |
| BACT000035 (*rplF*) | 4235 | 531 | 352510 | 353040 | species: *Lactobacillus johnsonii* [n=12] |
| BACT000036 (*rplL*) | 258 | 363 | 443413 | 443775 | species: *Lactobacillus gasseri* [n=55]; *Lactobacillus johnsonii* [n=48]; *Lactobacillus paragasseri* [n=17]; *Lactobacillus* sp. [n=2] |
| BACT000038 (*rplI*) | 5465 | 456 | 13144 | 13599 | species: *Lactobacillus johnsonii* [n=13] |
| BACT000039 (*rplJ*) | 435 | 501 | 442869 | 443369 | species: *Lactobacillus johnsonii* [n=46] |
| BACT000040 (*rplK*) | 651 | 426 | 439257 | 439682 | species: *Lactobacillus johnsonii* [n=29] |
| BACT000042 (*rplM*) | 271 | 444 | 362772 | 363215 | species: *Lactobacillus johnsonii* [n=51]; *Lactobacillus* sp. [n=1] |
| BACT000043 (*rplN*) | 243 | 369 | 350677 | 351045 | species: *Lactobacillus johnsonii* [n=61]; *Lactobacillus* sp. [n=1] |
| BACT000044 (*rplO*) | 279 | 441 | 354190 | 354630 | species: *Lactobacillus johnsonii* [n=60]; *Lactobacillus* sp. [n=1] |
| BACT000045 (*rplP*) | 763 | 438 | 349730 | 350167 | species: *Lactobacillus johnsonii* [n=26] |
| BACT000046 (*rplQ*) | 266 | 384 | 358822 | 359205 | species: *Lactobacillus johnsonii* [n=17] |
| BACT000047 (*rplR*) | 3683 | 360 | 353068 | 353427 | species: *Lactobacillus johnsonii* [n=18] |

**Supplementary Table 4** Species identification by Ribosomal Multilocus Sequence Typing (rMLST) in public databases for molecular typing and microbial genome diversity (PubMLST) (cont.)

| Locus | Allele | Length | Start position | End position | Linked data values |
| --- | --- | --- | --- | --- | --- |
| BACT000048 (*rplS*) | 32647 | 351 | 768559 | 768909 | species: *Lactobacillus johnsonii* [n=1] |
| BACT000049 (*rplT*) | 576 | 357 | 1432326 | 1432682 | species: *Lactobacillus johnsonii* [n=31]; *Lactobacillus* sp. [n=1] |
| BACT000051 (*rplV*) | 559 | 354 | 348691 | 349044 | species: *Lactobacillus johnsonii* [n=31]; *Lactobacillus* sp. [n=1] |
| BACT000052 (*rplW*) | 3520 | 297 | 347209 | 347505 | species: *Lactobacillus johnsonii* [n=6] |
| BACT000053 (*rplX*) | 267 | 240 | 351066 | 351305 | species: *Lactobacillus johnsonii* [n=56]; *Lactobacillus* sp. [n=1] |
| BACT000056 (*rpmA*) | 15502 | 297 | 718193 | 718489 | species: *Lactobacillus johnsonii* [n=7] |
| BACT000057 (*rpmB*) | 413 | 186 | 738132 | 738317 | species: *Lactobacillus johnsonii* [n=63]; *Lactobacillus* sp. [n=1] |
| BACT000058 (*rpmC*) | 216 | 198 | 350157 | 350354 | species: *Lactobacillus johnsonii* [n=63]; *Lactobacillus gasseri* [n=51]; *Lactobacillus paragasseri* [n=22]; *Lactobacillus* sp. [n=2] |
| BACT000059 (*rpmD*) | 228 | 183 | 353982 | 354164 | species: *Lactobacillus johnsonii* [n=62]; *Lactobacillus taiwanensis* [n=25]; *Lactobacillus* sp. [n=1] |
| BACT000060 (*rpmE*) | 278 | 252 | 306407 | 306658 | species: *Lactobacillus johnsonii* [n=36] |
| BACT000061 (*rpmF*) | 242 | 192 | 1140889 | 1141080 | species: *Lactobacillus johnsonii* [n=59]; *Lactobacillus gasseri* [n=9]; *Lactobacillus paragasseri* [n=2]; *Lactobacillus* sp. [n=1] |
| BACT000062 (*rpmG*) | 316 | 150 | 1410674 | 1410823 | species: *Lactobacillus johnsonii* [n=62]; *Lactobacillus gasseri* [n=58]; *Lactobacillus taiwanensis* [n=27]; *Lactobacillus paragasseri* [n=20]; *Lactobacillus* sp. [n=2] |

**Supplementary Table 4** Species identification by Ribosomal Multilocus Sequence Typing (rMLST) in public databases for molecular typing and microbial genome diversity (PubMLST) (cont.)

| Locus | Allele | Length | Start position | End position | Linked data values |
| --- | --- | --- | --- | --- | --- |
| BACT000062 (*rpmG*) | 762 | 150 | 438152 | 438301 | species: *Lactobacillus johnsonii* [n=38] |
| BACT000063 (*rpmH*) | 523 | 141 | 1872534 | 1872674 | species: *Lactobacillus johnsonii* [n=52]; *Lactobacillus* sp. [n=1] |
| BACT000064 (*rpmI*) | 255 | 201 | 1432715 | 1432915 | species: *Lactobacillus johnsonii* [n=60]; *Lactobacillus gasseri* [n=58]; *Lactobacillus taiwanensis* [n=26]; *Lactobacillus paragasseri* [n=19]; *Lactobacillus* sp. [n=2] |
| BACT000065 (*rpmJ*) | 223 | 117 | 356900 | 357016 | species: *Lactobacillus johnsonii* [n=61]; *Lactobacillus gasseri* [n=58]; *Lactobacillus taiwanensis* [n=24]; *Lactobacillus paragasseri* [n=22]; *Lactobacillus* sp. [n=2] |

**Supplementary Table 5** Unique genes of *L. johnsonii* KD1 generated by comparing against 20 other *L. johnsonii* strains through the protein family sorter tool with PATRIC genus-specific families (PLfams) in BV-BRC version 3.25.3.

| **CDS** | **Position (bp)** | **Length (bp)** |
| --- | --- | --- |
| Type I restriction-modification system, specificity subunit S | 1,111,551-1,112,621 | 1,071 |
| Integrase | 1,114,507-1,115,442 | 936 |
| Late competence protein ComEA, DNA receptor | 1,190,722-1,191,408 | 687 |
| Uncharacterized deacetylase | 1,470,768-1,471,910 | 1,143 |
| Type I restriction-modification system, specificity subunit S | 1,540,690-1,541,892 | 1,203 |
| Type I restriction-modification system, DNA-methyltransferase subunit M | 1,541,879-1,543,495 | 1,617 |
| Type I restriction-modification system, restriction subunit R | 1,543,495-1,546,560 | 3,066 |
| hypothetical protein | 406,316-412,822 | 6,507 |
| hypothetical protein | 804,817-805,263 | 447 |

**Supplementary Table 6** Annotated genes coding for a type I restriction-modification system in *L. johnsonii* KD1 using BV-BRC version 3.25.3

| **CDS** | **Position (bp)** | **Length (bp)** |
| --- | --- | --- |
| Type I restriction-modification system, specificity subunit S | 1,111,551-1,112,621 | 1,071 |
| Type I restriction-modification system, specificity subunit S | 1,112,635-1,113,816 | 1,182 |
| Type I restriction-modification system, specificity subunit S | 1,115,513-1,116,736 | 1,224 |
| Type I restriction-modification system, DNA-methyltransferase subunit M (EC 2.1.1.72) | 1,116,729-1,118,372 | 1,644 |
| Type I restriction-modification system, restriction subunit R (EC 3.1.21.3) | 1,118,386-1,121,487 | 3,102 |
| Type I restriction-modification system, specificity subunit S | 1,540,690-1,541,892 | 1,203 |
| Type I restriction-modification system, DNA-methyltransferase subunit M (EC 2.1.1.72) | 1,541,879-1,543,495 | 1,617 |
| Type I restriction-modification system, restriction subunit R (EC 3.1.21.3) | 1,543,495-1,546,560 | 3,066 |

**Supplementary Table 7** CRISPR-Cas system in *L. johnsonii* KD1 detected using the CRISPRCasFinder tool version 4.2.30

| **Element** | **Position** | **Spacer (n)** | **Direct repeat (DR) (n)** | **DR sequence** |
| --- | --- | --- | --- | --- |
| CRISPR | 1,158,738-1,158,952 | 3 | 4 | TTAGGATCACCTCCACATACGTGGAGAATACA |

**Supplementary Table 8** *L. johnsonii* KD1 specific pathways and respective protein families. Pathways, constituent proteins, and respective enzymatic products identified via the BV-BRC comparative systems/ pathways by comparing *L. johnsonii* KD1 to two *L. plantarum* ATCC14917 genomes

| **Pathway Name** | **Pathway Class** | **Protein family** | **Product** |
| --- | --- | --- | --- |
| gamma-Hexachlorocyclohexane degradation | Xenobiotics Biodegradation and Metabolism | 2-haloalkanoic acid dehalogenase (EC 3.8.1.2) | Glycolate (acetic acid) |
| 2,4-Dichlorobenzoate degradation | Xenobiotics Biodegradation and Metabolism | IAA acetyltransferase (EC 2.3.1.-) | Benzoyl-CoA |
| Geraniol degradation | Xenobiotics Biodegradation and Metabolism | 3-ketoacyl-CoA thiolase (EC 2.3.1.16) @ Acetyl-CoA acetyltransferase (EC 2.3.1.9) | 3-methylcrotonyl-CoA |
| Ethylbenzene degradation | Xenobiotics Biodegradation and Metabolism | IAA acetyltransferase (EC 2.3.1.-) & 3-ketoacyl-CoA thiolase (EC 2.3.1.16) @ Acetyl-CoA acetyltransferase (EC 2.3.1.9) | Benzoyl-CoA and Acetyl-CoA |
| Bisphenol A degradation | Xenobiotics Biodegradation and Metabolism | 2,5-diketo-D-gluconic acid reductase (EC 1.1.1.-) (x2) | 4'-hydroxyacetophenone |
| Ether lipid metabolism | Lipid Metabolism | IAA acetyltransferase (EC 2.3.1.-) | Plasmanic acid |
| C21-Steroid hormone metabolism | Lipid Metabolism | 2,5-diketo-D-gluconic acid reductase (EC 1.1.1.-) (x2) | 11-dehydrocorticosterone |
| Linoleic acid metabolism | Lipid Metabolism | 2,5-diketo-D-gluconic acid reductase (EC 1.1.1.-) (x2) | 13-OXDE (Linoleic acid analogue) |
| Fatty acid elongation in mitochondria | Lipid Metabolism | 3-ketoacyl-CoA thiolase (EC 2.3.1.16) @ Acetyl-CoA acetyltransferase (EC 2.3.1.9) | Fatty acid precursor |
| Diterpenoid biosynthesis | Biosynthesis of Secondary Metabolites | IAA acetyltransferase (EC 2.3.1.-) | Taxol |
| Biosynthesis of type II polyketide backbone | Biosynthesis of Polyketides and Nonribosomal Peptides | 2,5-diketo-D-gluconic acid reductase (EC 1.1.1.-) (x2) and IAA acetyltransferase (EC 2.3.1.-) | Polyketide intermediates |

**Supplementary Table 9** Sixteen viral proteins were found in *L. johnsonii* KD1.

| **Query_id** | **Combined_Products** | **EC No.** | **Function / Pathway** | **Group** |
| --- | --- | --- | --- | --- |
| fig\|1578.2103.peg.115\| | HIV-1 protein Vpu | N/A | HIV protein also involved in signaling | Viral |
| fig\|1578.2103.peg.118\| | Hepadnaviridae DNA polymerase / reverse transcriptase / ribonuclease H [EC:2.7.7.7 2.7.7.49 3.1.26.4] | [EC:2.7.7.7 2.7.7.49 3.1.26.4] | Hepatitis replication and signaling | Viral |
| fig\|1578.2103.peg.191\| | Hepadnaviridae DNA polymerase / reverse transcriptase / ribonuclease H [EC:2.7.7.7 2.7.7.49 3.1.26.4] | [EC:2.7.7.7 2.7.7.49 3.1.26.4] | Hepatitis replication and signaling | Viral |
| fig\|1578.2103.peg.638\| | Lymphocryptovirus latent membrane protein 1 | N/A | Epstein-Barr virus infection, Viral carcinogenesis | Viral |
| fig\|1578.2103.peg.904\| | Cyanophage dATP/dGTP diphosphohydrolase [EC:3.6.1.-] | [EC:3.6.1.-] | Purine metabolism / viral | Viral |
| fig\|1578.2103.peg.907\| | Lymphocryptovirus latent membrane protein 1 | N/A | Epstein-Barr virus infection, Viral carcinogenesis | viral |
| fig\|1578.2103.peg.916\| | Escherichia phage polynucleotide kinase [EC:2.7.1.78 3.1.3.34] | [EC:2.7.1.78 3.1.3.34] | Kinase, viral | Viral |
| fig\|1578.2103.peg.1072\| | Hepadnaviridae DNA polymerase / reverse transcriptase / ribonuclease H [EC:2.7.7.7 2.7.7.49 3.1.26.4] | [EC:2.7.7.7 2.7.7.49 3.1.26.4] | Hepatitis replication and signaling | Viral |

**Supplementary Table 9** Sixteen viral proteins were found in *L. johnsonii* KD1 (cont.).

| fig\|1578.2103.peg.1082\| | Orthopoxvirus virion membrane protein A21 | N/A | Orthopoxvirus virion membrane protein A21 | Viral |
| --- | --- | --- | --- | --- |
| fig\|1578.2103.peg.1097\| | Orthonairovirus envelopment polyprotein | N/A | Orthonairovirus envelope polyprotein | Viral |
| fig\|1578.2103.peg.1102\| | Mastadenovirus hexon-interlacing protein | N/A | Viral structural protein | Viral |
| fig\|1578.2103.peg.1121\| | HTLV-1 accessory protein p30II | N/A | Viral genome structure and infection (inhibition) | Viral |
| fig\|1578.2103.peg.1154\| | Poxviridae cell surface-binding protein | N/A | Viral protein | Viral |
| fig\|1578.2103.peg.1305\| | Phenuiviridae envelopment polyprotein | N/A | Phenuiviridae envelopment polyprotein | Viral |
| fig\|1578.2103.peg.1528\| | HCMV TNF alpha-like receptor UL144 | N/A | Viral protein that regulates host immune system | Viral |
| fig\|1578.2103.peg.1601\| | Lymphocryptovirus latent membrane protein 1 | N/A | Epstein-Barr virus infection, Viral carcinogenesis | Viral |

**Supplementary Table 10** *L. johnsonii* KD1 genes potentially influencing survival in shrimp gut conditions and contributing to anti-viral activities

| **CDS** | **Positions (bp)** | **Length (bp)** | **Function** |
| --- | --- | --- | --- |
| Na(+)/H(+) antiporter | 127,397-128,968  188,672-190,498  1,484,623-1,485,999  1,801,104-1,802,264 | 1,572  1,827  1,377  1,161 | Exclude sodium outside cells |
| Potassium transport system protein Kup1  Potassium transport system protein Kup2 | 175,229-177,281  177,429-179,427 | 2,052  1,998 | Influx of potassium during hyperosmotic stress |
| Transcriptional regulator, LysR family  RNA polymerase sigma factor RpoD | 62,004-62,931  885,027-886,146 | 927  1,119 | Regulators |
| Choloylglycine hydrolases | 68,977-69,928  837,986-838,967  1,046,693-1,047,671 | 951  981  978 | Resistance to bile salts |
| Heat shock proteins  GroES,  GroEL,  GrpE  DnaK  DnaJ | 485,395-485,679  485,710-487,341  812,557-813,135  813,153-815,027  815,107-816,273 | 285  1,632  579  1,875  1,167 | Repair of damaged proteins |
| Clp proteases  ClpP  ClpE  ClpA  ClpC  ClpX | 1,331,592-1,332,179  1,389,372-1,391,540  1,782,552-1,784,666  331,408-333,876  1,179,627-1,180,893 | 588  2,169  2,115  2,469  1,266 | Refolding or degrading denatured proteins |

Supplementary Table 10 *L. johnsonii* KD1 genes potentially influencing survival in shrimp gut conditions and contributing to anti-viral activities (cont.)

| **CDS** | **Positions (bp)** | **Length (bp)** | **Function** |
| --- | --- | --- | --- |
| UvrABC system protein B  ATP-dependent DNA helicase UvrD/PcrA | 1,338,677-1,340,693  1,508,100-1,510,346 | 2,016  2,247 | DNA repair |
| D-lactate dehydrogenase  L-lactate dehydrogenase | 52,591-53,604  312,112-313,083  849,932-850,858  1,530,188-1,531,096  1,836,707-1,837,945 | 1,014  972  927  909  1239 | Lactic acid production |
| LytR-transcriptional regulator (*epsA*)  Polymerization and chain length determination protein (*epsB*)  Tyrosine kinase (*epsC*)  Protein-tyrosine-phosphate phosphohydrolase (*epsD*)  Undecaprenyl-phosphate galactosephosphotransferase (*epsE*)  UDP-galactopyranase mutase (*glf*)  Oligosaccharide translocase (*epsU*)  Glycosyltransferases | 1,171,218-1,172,223  1,170,348- 1,171,212  1,169,576-1,170,338  1,168,799-1,169,570  1,168,112-1,168,772  1,160,594-1,161,713  1,159,161-1,160,589  1,163,038-1,163,962  1,163,979-1,165,074  1,165,080-1,166,202  1,166,198-1,167,299  1,167,325-1,168,102 | 1,005  864  762  771  660  1,119  1,428  924  1,095  1,122  1,101  777 | Exopolysaccharide production |

Supplementary Table 10 *L. johnsonii* KD1 genes potentially influencing survival in shrimp gut conditions and contributing to anti-viral activities (cont.)

| **CDS** | **Positions (bp)** | **Length (bp)** | **Function** |
| --- | --- | --- | --- |
| Fructosyltransferase | 1,288,765-1,291,003 | 2,238 | Levansucrase/ Inulosucrase |
| Homolog of bacteriocin helveticin-J | 558,404-559,462 | 1,056 | - |
| Holin homolog | 622,460-633,587 | 11,128 | - |

**Supplementary Table 11** Oligonucleotides used in this study following previous study [6]

| **Oligo name** | **Sequence (5’-3’)** | **Function** |
| --- | --- | --- |
| WSSV229_F | GATGGAAACGGTAACGAATCTGAA | Quantitation of WSSV copies by qPCR |
| WSSV447_R | CAGAGCCTAGTCTATCAATCAT |  |
| Actin_F | CCTCGCTGGAGAAGTCCTAC | Internal control for shrimp DNA extraction |
| Actin_R | TGGTCCAGACTCGTCGTACTC |  |
| Perox-F | GTGAACGGTAGTCCTTTACCTAAT | Peroxinectin (PX) |
| Perox-R | CGAGGTCCATAGAAAGCATCTC |  |
| SP-F | CCGTCTTGGAGAATACGACTTGAG | Serine proteases (SP) |
| SP-R | GCTACAGGTAGGCTGGATAACTTG |  |
| Propo1-F | CAAGCCCTTCGACTACCATATAC | Prophenoloxidase-1 (proPO I) |
| Propo1-R | CTGACTGTTCACTTGAGTTCCC |  |
| ALF1-F | TTACTTCAATGGCAGGATGTGG | Anti-lipopolysaccharide factor-1(ALF-1) |
| ALF1-R | GTCCTCCGTGATGAGATTACTCTG |  |

**Supplementary Table 12** Raw data from Illumina and Nanopore sequencing

| **Data** | **Illumina** | **Nanopore** |
| --- | --- | --- |
| Total bases (bp) | 2,907,555,736 | 487,971,249.0 |
| Read length N50 (bp) | - | 5,722.0 |
| Number of reads | 19,255,336 | 118,488.0 |
| Median read length (bp) | - | 2,761.0 |
| Mean read length (bp) | 151 | 4,118.3 |
| Genome coverage | 1,551X | 261X |

**
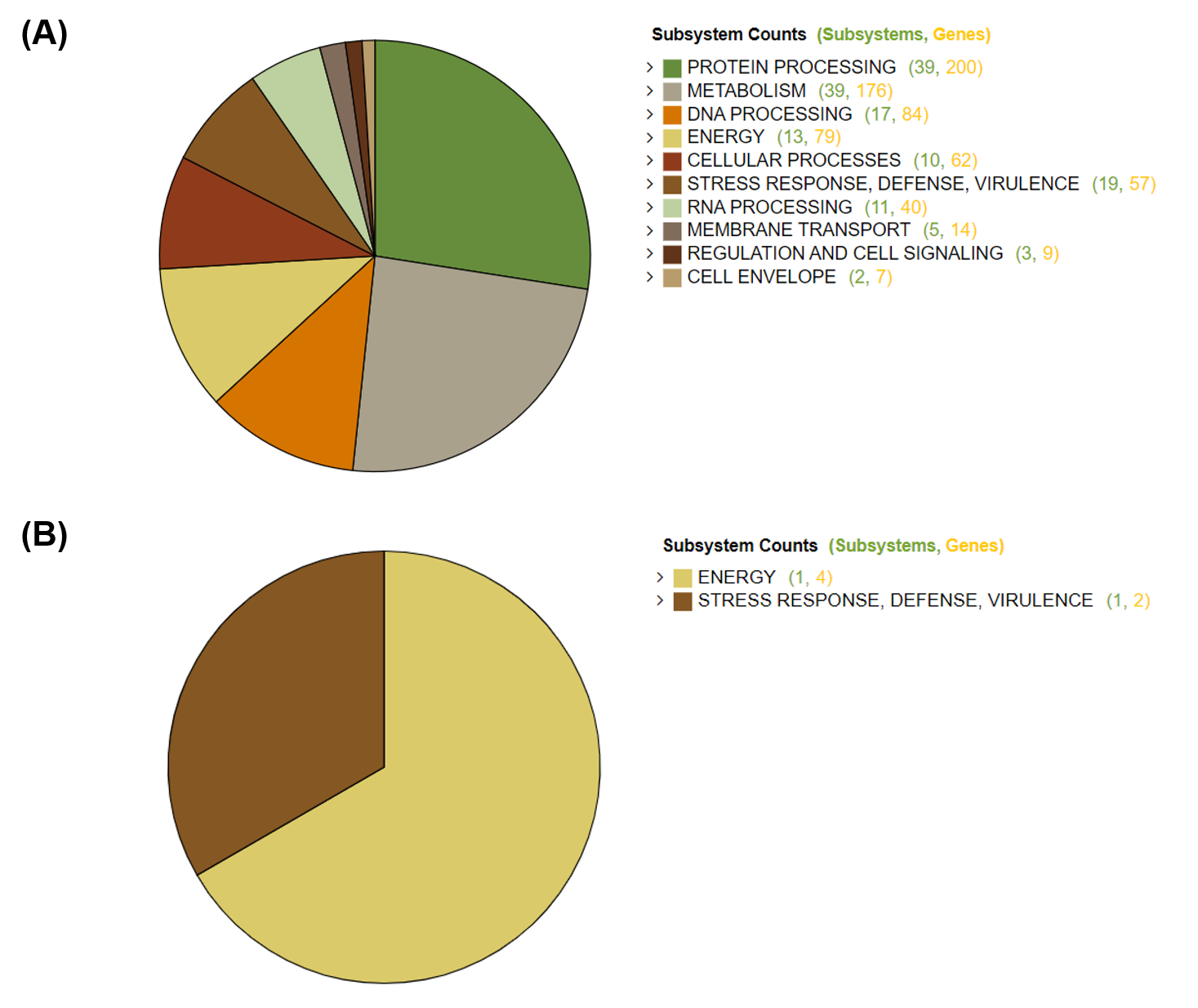
**

**Supplementary Fig. 1** Pie charts of subsystem category distribution in chromosome (A) and plasmid (B) of *L. johnsonii* KD1

**Supplementary Fig. 2** Efficacy of two probiotics of *L. johnsonii* KD1 in this study and *L. plantarum* ATCC 14917 that was investigated in our previous study [6] on shrimp WSSV inhibition. Mean of percentage cumulative mortality ± standard error in shrimps submerged with 4.5 × 10^10^ [100X] CFU/ 2 L seawater of each probiotic for five days with n = 30 shrimps per replicate (three replicates/ group) while the negative and positive groups without probiotic treatments with n = 30 shrimps per replicate (three replicates/ group) followed by WSSV infection except the negative group. Corresponding day post infection (dpi.) at p < 0.05 based on ANOVA was tested by Duncan’s test, represented by small alphabets (a, b, and c). Asterisks (*) indicate significant differences between the treatment and the positive control on corresponding dpi. at p < 0.05 based on independent sample T-test and Mann-Whitney Test.

**
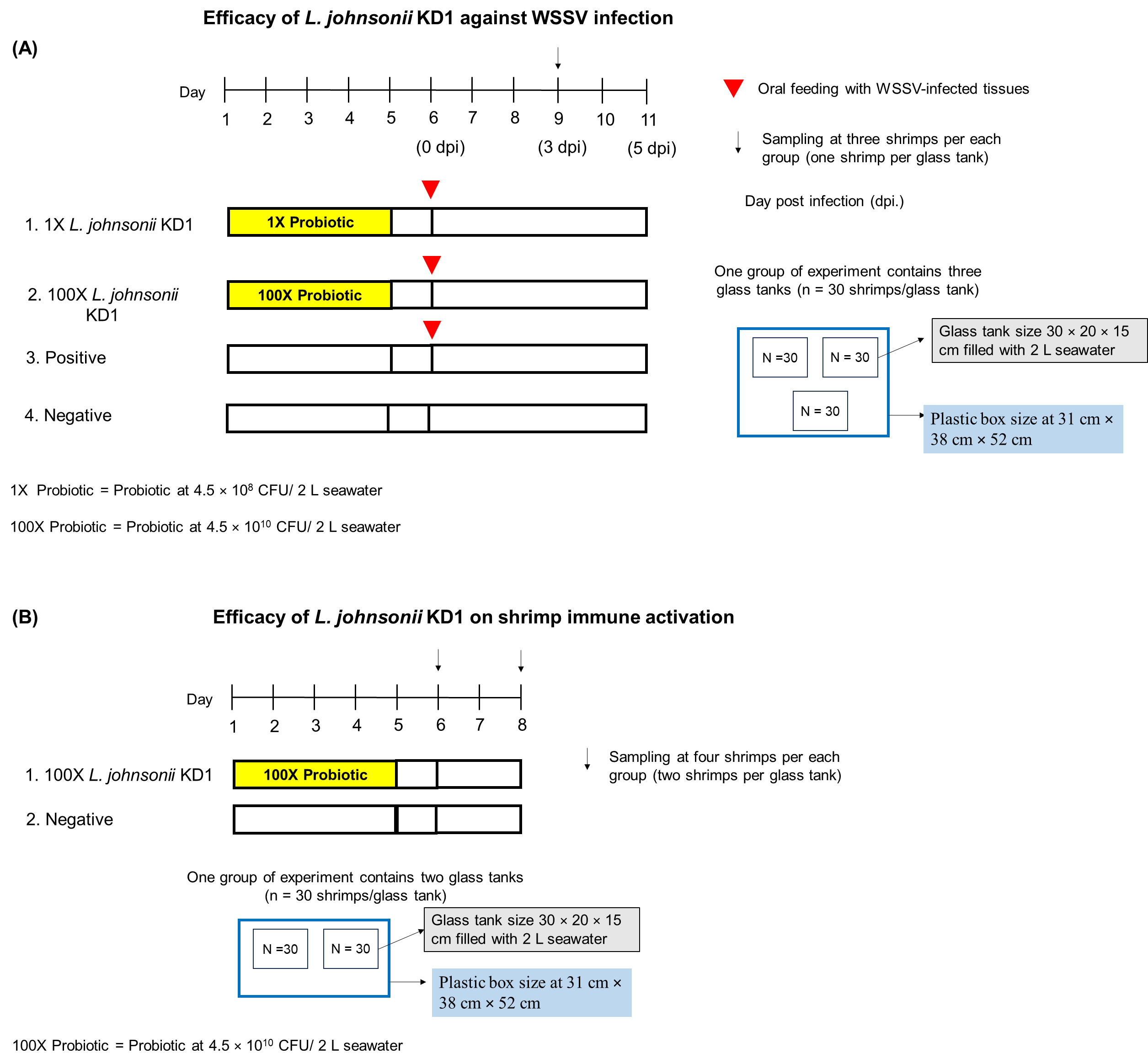
**

**Supplementary Fig. 3** Experimental design (A) Efficacy of *L. johnsonii* KD1 against WSSV infection. Four experimental groups consisted of group 1: shrimps treated with 1X *L. johnsonii* KD1 (4.5 × 10^8^ CFU/2L seawater) for five days followed by WSSV infection, group 2: shrimps treated with 100X *L. johnsonii* KD1 (4.5 × 10^10^ CFU/2L seawater) for five days followed by WSSV infection, group 3: shrimps treated with WSSV infection, and group 4: shrimps without any treatments. Each group contained three glass tanks, each sized 30 × 20 × 15 cm and filled with 2 L seawater containing 30 shrimps/tank. The three tanks were placed into a plastic box sized 31 × 38 × 52 cm in order to avoid contamination among groups. Three shrimp samples were collected at 3 days post infection (3 dpi.) to quantify WSSV load by qPCR and shrimp mortality was observed until at 5 dpi. (B) Efficacy of *L. johnsonii* KD1 on shrimp immune activation. Two experimental groups consisted of group 1: shrimps treated with 100X *L. johnsonii* KD1 (4.5 × 10^10^ CFU/2L seawater) for five days and group 2: shrimps without any treatments. Each group contain two glass tanks, each sized 30 × 20 × 15 cm and filled with 2 L seawater containing 30 shrimps/tank. The two tanks were placed into plastic box sized 31 × 38 × 52 cm in order to avoid contamination between groups. Four shrimp samples were collected at day 6 (or one day post-probiotics regimen in treated groups) and day 8 (or three days post-probiotics regimen).

**Reference**

Dekham K, Jitrakorn S, Charoonnart P, Isarangkul D, Chaturongakul S, Saksmerprome V: Probiotics expressing double-stranded RNA targeting VP28 efficiently protect shrimps from WSSV infection. Aquac Rep. 2022;23:101067.
